# Description of *Heterorhabditis americana* n. sp. (Rhabditida, Heterorhabditidae), a new entomopathogenic nematode species isolated in North America

**DOI:** 10.1186/s13071-025-06702-5

**Published:** 2025-03-11

**Authors:** Ricardo A. R. Machado, Joaquín Abolafia, María-Cristina Robles, Alba N. Ruiz-Cuenca, Aashaq Hussain Bhat, Ebrahim Shokoohi, Vladimír Půža, Xi Zhang, Matthias Erb, Christelle A. M. Robert, Bruce Hibbard

**Affiliations:** 1 Experimental Biology Research Group, Institute of Biology, Faculty of Sciences, University of Neuchâtel, Neuchâtel, Switzerland; 2 Departamento de Biología Animal, Biología Vegetal y Ecología, Universidad de Jaén, Campus ‘Las Lagunillas’, Jaén, Spain; 3 University Centre for Research and Development and Department of Bioscience, Chandigarh University, Mohali, 140413 Punjab India; 4 Department of Biochemistry, Microbiology and Biotechnology, University of Limpopo, Sovenga, 0727 South Africa; 5 Institute of Entomology, Biology Centre of the Czech Academy of Sciences, CAS, 37005 České Budějovice, Czech Republic; 6 Faculty of Agriculture and Technology, University of South Bohemia, 37005 České Budějovice, Czech Republic; 7 Institute of Plant Sciences, University of Bern, Bern, Switzerland; 8 Plant Genetics Research Unit, United States Department of Agriculture (USDA)-Agricultural Research Service, Columbia, MO USA

**Keywords:** Entomopathogenic nematodes, Biocontrol agents, Species description, Nematode morphology, Phylogenetics, Taxonomy, *Photorhabdus*

## Abstract

**Background:**

*Heterorhabditis* are important biological control agents in agriculture. Two *Heterorhabditis* populations, S8 and S10, were isolated from agricultural soils in the United States of America. Molecular analyses, based on mitochondrial and nuclear genes, showed that these populations are conspecific and represent a novel species of the “*Bacteriophora*” clade. This species was named *Heterorhabditis americana* n. sp. and is described in this study.

**Methods:**

To describe *H. americana* n. sp., we carried out phylogenetic reconstructions using multiple genes, characterized their morphology, conducted self-crossing and cross-hybridization experiments, and isolated and identified their symbiotic bacteria.

**Results:**

*Heterorhabditis americana* n. sp. is molecularly and morphologically similar to *H. georgiana*. Morphological differences between the males of *H. americana* n. sp. and *H. georgiana* include variations in the excretory pore position, the gubernaculum size, the gubernaculum-to-spicule length ratio, the tail length, and the body diameter. Infective juveniles (IJs) of *H. americana* n. sp. differ from *H. georgiana* IJs because *H. americana* n. sp. IJs have an invisible bacterial cell pouch posterior to the cardia and a small posterior phasmid, whereas *H. georgiana* IJs have a visible bacterial cell pouch and an inconspicuous phasmid. Hermaphrodites of *H. americana* n. sp. and *H. georgiana* are differentiated by the body length, the nerve ring distance from the anterior end, the excretory pore distance from the anterior end, the anal body diameter, and the c′ ratio. Females of *H. americana* n. sp. can be differentiated from *H. georgiana* females by the anal body diameter and the c′ ratio. Reproductive isolation was confirmed, as *H. americana* n. sp. does not produce viable offspring with any of the species of the “*Bacteriophora*” clade. *Heterorhabditis americana* n. sp. is associated with the symbiotic bacterium *Photorhabdus kleinii*.

**Conclusions:**

Based on the observed morphological and morphometric differences, the distinct phylogenetic placement, and the reproductive isolation, the nematode isolates S8 and S10 represent a novel species, which we named *Heterorhabditis americana* n. sp. This study provides a detailed characterization of this novel species, contributing to enhancing our knowledge of species diversity and evolutionary relationships of the *Heterorhabditis* genus.

**Graphical Abstract:**

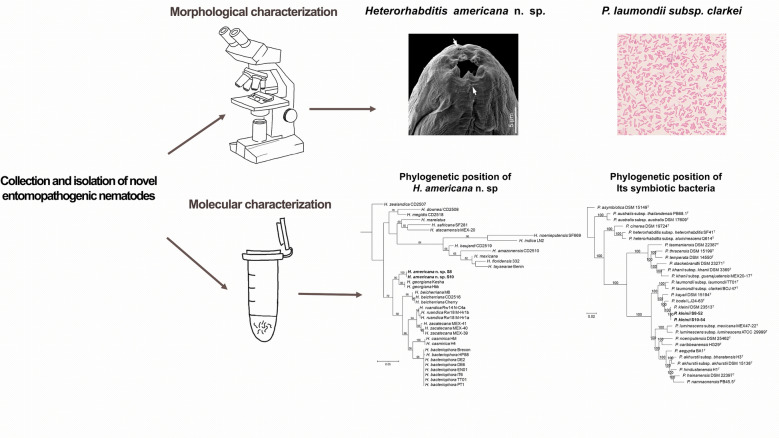

**Supplementary Information:**

The online version contains supplementary material available at 10.1186/s13071-025-06702-5.

## Background

Entomopathogenic nematodes (EPNs) of the genus *Heterorhabditis* Poinar (1976) are soil-dwelling organisms that parasitize small arthropods, particularly insects [1]. These nematodes maintain an obligate mutualistic relationship with entomopathogenic bacteria (EPB) of the genus *Photorhabdus* [2, 3]. The nematodes colonize their host through natural openings (mouth, spiracles, or anus), or by directly penetrating the cuticle. Inside the host, the EPNs release their symbiotic bacterial partners [4, 5]. The bacteria then proliferate and release immunosuppressive compounds, lytic enzymes, and toxins, leading to death of the host [6–9]. The insects tissues serve as a nutrient rich source, in which the nematodes grow and reproduce. Upon resource depletion, the nematode re-establish symbiosis with *Photorhabdus* bacteria and abandon the depleted insect cadaver in search of a new host [10]. Due to the ability to rapidly kill their host, this lethal symbiotic pair is used for the biocontrol of agricultural pests [11–15].

There is still some controversy regarding the number of valid species of *Heterorhabditis* [16–20]. Most of the described species are currently considered valid species, while others have been synonymized, reinstated as valid species, or proposed as *species inquirendae* [16, 17, 19, 21–23]. More specifically, *Heterorhabditis heliothidis* (Khan, Brooks & Hirschmann, 1976) Poinar, Thomas & Hess, 1977, previously synonymized with *H. bacteriophora*, was proposed as *species inquirenda*, and *H. egyptii* Abd-Elgawad & Ameen (2005) and *H. hambletoni* Pereira (1937) were reinstated as valid species [22]. Moreover, *H. brevicaudis*, *H. gerrardi*, *H. hawaiiensis*, *H. pakistanensis*, *H. somsookae*, and *H. sonorensis* were synonymized [16, 17, 23]. Hence, the genus *Heterorhabditis* contains at least 22 species that are currently considered valid [17, 22, 24–43]. Two of them, *H. egyptii* and *H. hambletoni*, lack molecular data and cannot be included in molecular or phylogenetic studies. An additional species, *Heterorhabditis alii*, has recently been proposed, although morphological and molecular support for its novel taxonomic status is lacking [44]. It is therefore proposed here as a *species inquirenda.*

It is important to note that the primary argument supporting the proposals to synonymize several *Heterorhabditis* species was the small differences observed in the internal transcribed spacer (ITS) sequences [16, 17]. However, due to the limited phylogenetic resolution power of ribosomal genes, additional molecular evidence, ideally from phylogenomic analyses of whole nuclear and/or mitochondrial genome data, should be incorporated to substantiate these synonymization proposals [18, 22, 24, 45]. In support of this, a recent phylogenomic study evaluated the molecular systematics value of multiple commonly used and several novel gene markers to resolve the taxonomy of the genus *Heterorhabditis*. The most robust and well-resolved phylogenetic relationships were reconstructed using both whole nuclear and mitochondrial genomes [45].

In this study, we characterized a novel entomopathogenic nematode species, *Heterorhabditis americana* n. sp., based on morphological and molecular evidence. The objectives of this study were (1) to reconstruct phylogenetic relationships using taxonomically relevant genes to confirm the distinct evolutionary lineage of this species, (2) to perform detailed morphological and morphometric analyses, (3) to carry out self-crossing and cross-hybridization experiments to test for the reproductive isolation of *H. americana* n. sp., and (4) to isolate and characterize the symbiotic bacteria associated with this novel nematode species. Our study contributes to the understanding of entomopathogenic nematode biodiversity and supports the development of sustainable pest management strategies in agriculture.

## Methods

### Nematode origin and rearing

Nematodes were isolated from soil samples by the insect baiting [46] and the White trap methods [47]. Soil samples to isolate *H. americana* n. sp. S8 were collected in the southeastern part of the state of Nebraska (decimal degree coordinates 40.8341, −96.6686), and the soil samples to isolate *H. americana* n. sp. S10 were collected in the state of South Dakota (decimal degree coordinates 43.9925, −96.7284).

### Morphological characterization

Hermaphrodites, amphimictic males, and amphimictic females were obtained by dissecting *Galleria mellonella* insects in Ringer’s solution [24]. Infective juveniles (IJs) were collected from White traps upon their emergence from *G. mellonella* cadavers [47]. Nematodes were fixed in 4% formaldehyde at 80 °C, then transferred to anhydrous glycerin, and mounted on permanent glass slides with extra paraffin wax layers to preserve their three-dimensional shape, as detailed by Grisse [48]. Morphological measurements were performed on an Olympus BX51 microscope (Tokyo, Japan) equipped with integrated software. Fifteen specimens at each developmental stage were morphologically characterized.

### Light microscopy (LM) and scanning electron microscopy (SEM)

Specimens for light microscopy (LM) and scanning electron microscopy (SEM) were prepared following the protocol of Abolafia [49]. Briefly, nematodes fixed in 4% formalin were transferred to anhydrous glycerin using the Siddiqi’s lactophenol-glycerin method [50]. The nematode specimens were then permanently mounted on glass slides following the glycerin-paraffin method [50, 51]. LM images were captured on a Nikon Eclipse 80i microscope (Olympus, Tokyo, Japan) equipped with differential interference contrast (DIC) optics and a Nikon Digital Sight DS-U1 camera. For SEM, nematodes stored in glycerin were rehydrated in distilled water (dH_2_O), dehydrated through a graded ethanol-acetone series, dried at a critical point using liquid CO_2_, mounted on SEM stubs with copper tape, coated with gold in a sputter coater, and imaged with a Zeiss Merlin microscope (5 kV) (Zeiss, Oberkochen, Germany) [52]. Images from LM and SEM were processed and merged using Adobe^®^ Photoshop^®^ CS (Microsoft Corporation, Redmond, WA, USA). Morphological characteristics of all valid *Heterorhabditis* species were compiled from original publications [17, 22, 24, 25, 27–39, 41, 42, 53–74]. Demanian indices and other morphological ratios were calculated according to de Man [75]. Stoma morphology was described following De Ley [76], and spicule and the gubernaculum structures were described following the terminology of Abolafia and Peña-Santiago [77].

### Self-crossing and cross-hybridization experiments

Self-crossing and cross-hybridization tests were conducted on lipid agar plates [78]. Given that *H. americana* n. sp. is morphologically and molecularly more similar to the species of the “*Bacteriophora*” clade, the following nematode species and isolates were included in these experiments: *H. americana* n. sp. S8 and S10, *H. bacteriophora* Brecon, *H. beicherriana* Cherry, *H. casmirica* HM, *H. georgiana* Kesha, *H. ruandica* Rw14_N-C4a, and *H. zacatecana* MEX-39. For each crossing type, 40 males and 40 virgin females were placed on 35 mm lipid agar plates and incubated at 25 ± 2 °C. The experiments followed a factorial design, with three replicates for each crossing type. Progeny production was recorded daily over 15 days. Each experiment was conducted three times under similar conditions.

### Nematode molecular characterization

Genomic DNA (gDNA) was extracted from approximately 10,000–20,000 IJs using the Norgen’s Genomic DNA Isolation Kit (Norgen Biotek Corp., Thorold, ON, Canada), following the manufacturer’s guidelines. The genomic regions amplified via polymerase chain reaction (PCR) included the mitochondrial cytochrome c oxidase I (*cox-1*) gene, the D2–D3 expansion segments of the 28S rRNA gene and the ITS region of the rRNA gene. The sequences of the primers used for PCR were evaluated in previous studies and are listed in Table S1 [16, 79–82]. PCR conditions are described in detail in our previous studies [22, 24, 83]. The PCR products were separated by electrophoresis (45 min at 100 V) on a 1% TBA (Tris–boric acid–ethylenediaminetetraacetic acid [EDTA]) buffered agarose gel stained with SYBR Safe DNA Gel Stain (Invitrogen, Carlsbad, CA, USA). Following electrophoresis, PCR products were purified using the FastGene Gel/PCR Extraction Kits (Nippon Genetics Co., Japan) and sequenced bidirectionally by Sanger sequencing (Microsynth AG, Balgach, Switzerland). Resulting sequences were manually curated, trimmed, and submitted to the National Center for Biotechnology Information (NCBI) database. Accession numbers are listed in Table S2.

### Intra-individual genetic diversity

To evaluate intra-individual genetic diversity, DNA was extracted from single virgin females. These females were individually washed with Ringer’s solution, and then with phosphate-buffered saline solution (PBS, pH 7.2), and transferred to sterile 0.5 ml Eppendorf tubes containing 20 μl of extraction buffer (17.7 μl nuclease-free dH_2_O, 2 μl 10× PCR buffer, 0.2 μl 1% Tween 20, and 0.1 μl proteinase K). Samples were frozen at −20 °C for 24 h, then incubated at 65 °C in a water bath for 1.2 h, followed by a 95 °C incubation for 10 min. Lysates were cooled on ice and centrifuged at 6000×*g* for 2 min. PCR and sequencing were carried out as described above.

### Phylogenetic relationships

Gene sequences used for the phylogenetic reconstructions were retrieved from the NCBI database using previously reported accession numbers [16, 22, 24] (Table S2). Phylogenetic relationships were reconstructed using the maximum likelihood method, based on the following nucleotide substitution models: Kimura 2-parameter (K2+G+I) for *cox-1* gene, Kimura 2-parameter (K2+G) for D2–D3 rRNA gene, and Tamura 3-parameter (TN92) for ITS rRNA gene. The best substitution models were determined through model-fit analysis in MEGA 7 [84–87]. Sequences were aligned using MUSCLE (v3.8.31) [88]. Phylogenetic trees with the highest log likelihood values, showing percentage clustering of taxa at branch points are shown. Initial trees for heuristic searches were obtained automatically by applying neighbor-joining and BioNJ algorithms to a matrix of pairwise distances estimated using the maximum composite likelihood (MCL) approach, and then selecting the topology with a superior log likelihood value. Evolutionary rate variations among sites were modeled with a discrete gamma distribution (+G), and some positions were treated as invariant (+I). The phylogenetic trees were graphically represented and edited using Interactive Tree of Life (v3.5.1) [89, 90].

### Symbiotic relationships

The *Photorhabdus* entomopathogenic bacteria associated with *H. americana* n. sp. S8 and *H. americana* n. sp. S10 nematodes were isolated following our established protocols [91, 92]. To this end, *G. mellonella* larvae (Lepidoptera: Pyralidae) were infested with 100 IJ nematodes. After 3–4 days, the insect cadavers were surface-sterilized and dissected with a surgical blade. Internal insect tissues were spread onto Luria–Bertani (LB) agar plates and incubated at 28 °C for 1–2 days. *Photorhabdus*-like colonies were subcultured to obtain monocultures. A single colony was selected and used for further experiments. Molecular identification was carried out based on whole-genome-based phylogenetic reconstructions and sequence similarity values [93]. Whole genomes were obtained as described [94]. Whole-genome sequence similarities were assessed through the digital DNA-DNA hybridization (dDDH) method using the recommended formula 2 of the genome-to-genome distance calculator (GGDC) [95–98].

## Results and discussion

**Order: Rhabditida Chitwood, 1933**

**Infraorder: Strongylida Weinland, 1858**

**Family: Heterorhabditidae Poinar, 1976**

**Genus:** ***Heterorhabditis*** **Poinar, 1976**

***Heterorhabditis americana*** **Machado, Abolafia, Robles, Ruiz-Cuenca, Bhat, Shokoohi, Půža, Zhang, Erb, Robert & Hibbard n. sp.**

***Type strain: Heterorhabditis americana*** n. sp. S8 is designated as the type strain of the species. An additional population, S10, of this species has been molecularly characterized in this study.

***Type specimens:*** A slide containing the male holotype (Mh-am-UL2025) and three slides with paratypes representing each developmental stage—hermaphroditic females (H-am-UL2025), males (M-am-UL2025), females (F-am-UL2025), and IJs (IJ-am-UL2025)—were deposited in the Nematology Collection at the University of Limpopo, South Africa. In addition, slides containing the following paratypes were deposited in the Nematology Collection at the University of Jaen, Spain: six hermaphroditic females (USA002-01 and USA002-02), four females (USA002-03), four males (USA002-04), four J2 juveniles (USA002-05), and four J3 juveniles (USA002-05).

***Type-host:*** The type host is unknown as the nematodes of this genus can be hosted by different insect species. *Heterorhabditis americana* n. sp. S8 and *H. americana* n. sp. S10 were isolated from soil samples using *Galleria* larvae as baits.

***Type-locality:*** *Heterorhabditis americana* n. sp. S8 was isolated from soil samples collected in the southeastern part of Nebraska (decimal degrees coordinates 40.8341, −96.6686), and *H. americana* n. sp. S10 was isolated from soil samples collected in South Dakota (decimal degrees coordinates 43.9925, −96.7284).

***Site in host:*** whole internal body.

***Representative DNA sequences:*** Representative sequences of *H. americana* n. sp. S8 were deposited in the GenBank database under the accession numbers PQ483104 (ITS), OK646639 (D2–D3), PQ483125 and PQ341163 (cytochrome c oxidase subunit I, *cox-1*), PQ367560 (NADH dehydrogenase subunit 4, *nad-4*), PQ367701 (fanconi-associated nuclease 1, *fan-1*), and PQ367748 (serine/threonine-protein phosphatase 4 regulatory subunit 1, *ppfr-1*). Representative sequences of *H. americana* n. sp. S10 include PQ483105 (ITS), MW817559 (D2–D3), PQ483126 and PQ341161 (cytochrome c oxidase subunit I, *cox-1*), PQ367558 (NADH dehydrogenase subunit 4, *nad-4*), PQ367699 (fanconi-associated nuclease 1, *fan-1*), and PQ367746 (serine/threonine-protein phosphatase 4 regulatory subunit 1, *ppfr-1*). Additional sequences are provided in previous literature [45].

***ZooBank registration:*** To comply with the regulations set out in Article 8.5 of the amended 2012 version of the International Code of Zoological Nomenclature (ICZN), details of the new species have been submitted to ZooBank. The Life Science Identifier (LSID) of the article is urn:lsid:zoobank.org:pub:36EBE371-1A95-4267-BE3C-91E264B27EA3. The LSID for the new name *H. americana* is urn:lsid:zoobank.org:act:0B2179A2-EE62-4D33-AB75-30DDFE8C7D66.

***Etymology:*** The specific name of this nematode species is derived from the continent where the specimens used for its description were collected (America).

## Description

Morphological and morphometric characteristics of *H. americana* n. sp. are presented in Figs. 1, 2, 3, 4, 5, 6, 7, 8, and 9 and in Tables 1, 2, 3, 4, and 5. Values for *H. americana* n. sp. are presented in bold.

**Fig. 1:**
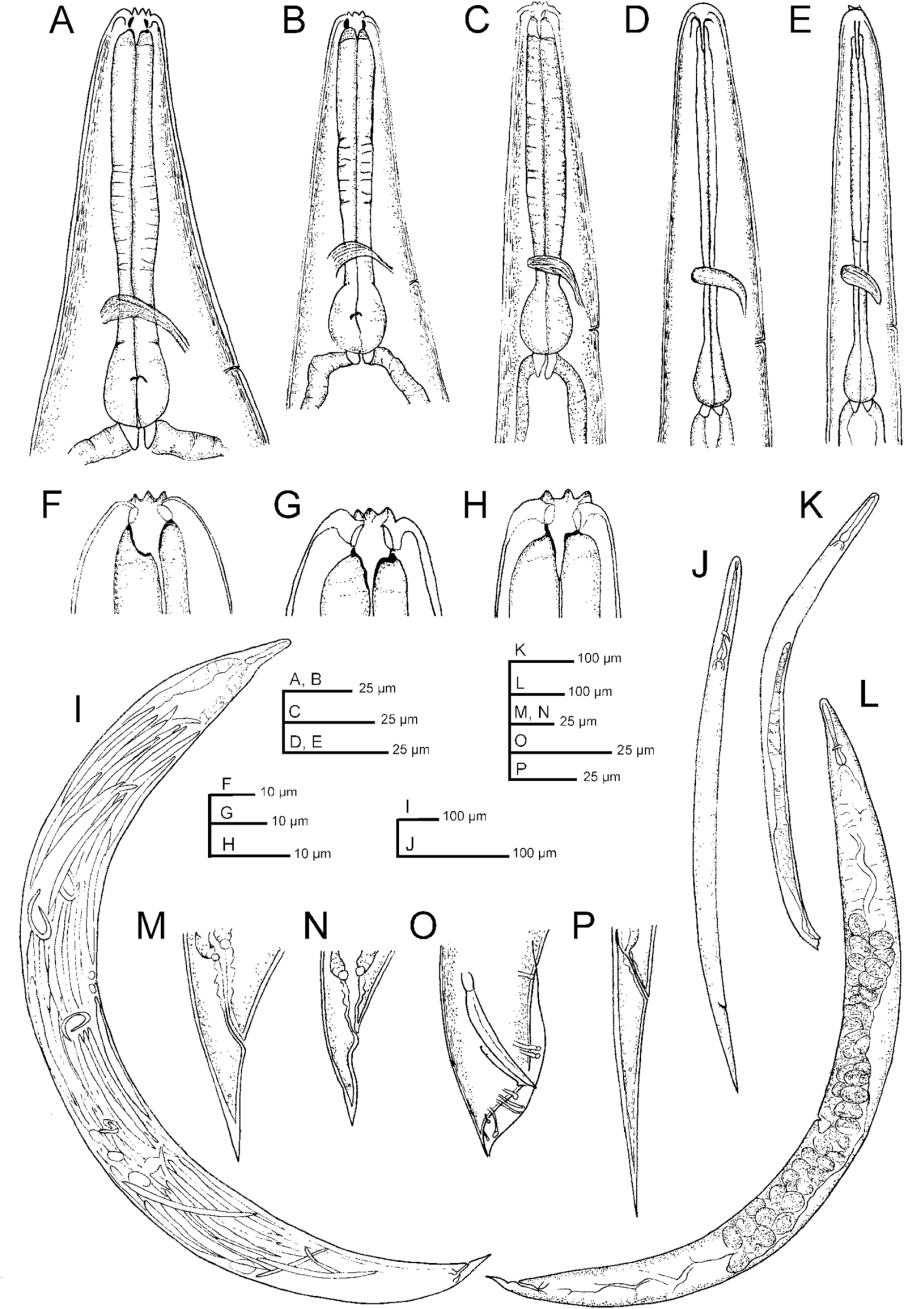
Line drawings of *H. americana* n. sp. S8. **A**–**E** Neck regions of a hermaphroditic female, a female, a male, a J2 infective juvenile, and a J3 infective juvenile, respectively. **F**–**H** Lip regions of a hermaphroditic female, a female, and a male, respectively. **I** A hermaphroditic female. **J** An infective juvenile. **K** An adult male. **L** An adult female. **M**–**P** Posterior ends of a hermaphroditic female, a female, a male, and a J2 infective juvenile, respectively

**Fig. 2:**
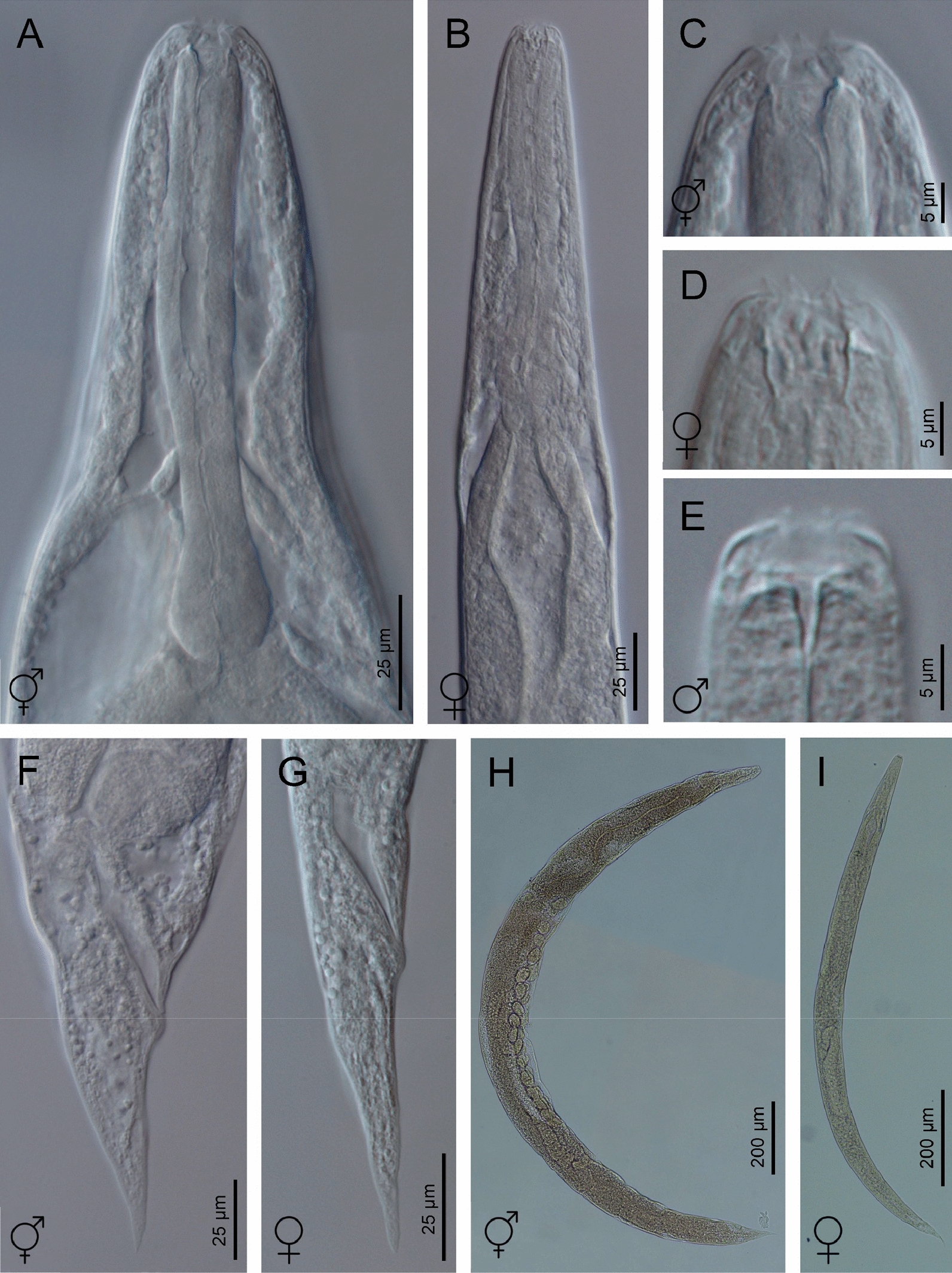
Light microscope (LM) photographs of *H. americana* n. sp. S8 hermaphroditic females and dioecious adults. **A**, **B** Pharyngeal regions of a hermaphroditic female and an adult female, respectively. **C**–**E** Lip regions of a hermaphroditic female, an adult female, and an adult male, respectively. **F**, **G** Posterior ends of a hermaphroditic female and a female adult, respectively. **H**, **I** Entire bodies of a hermaphroditic female and a female adult, respectively

**Fig. 3:**
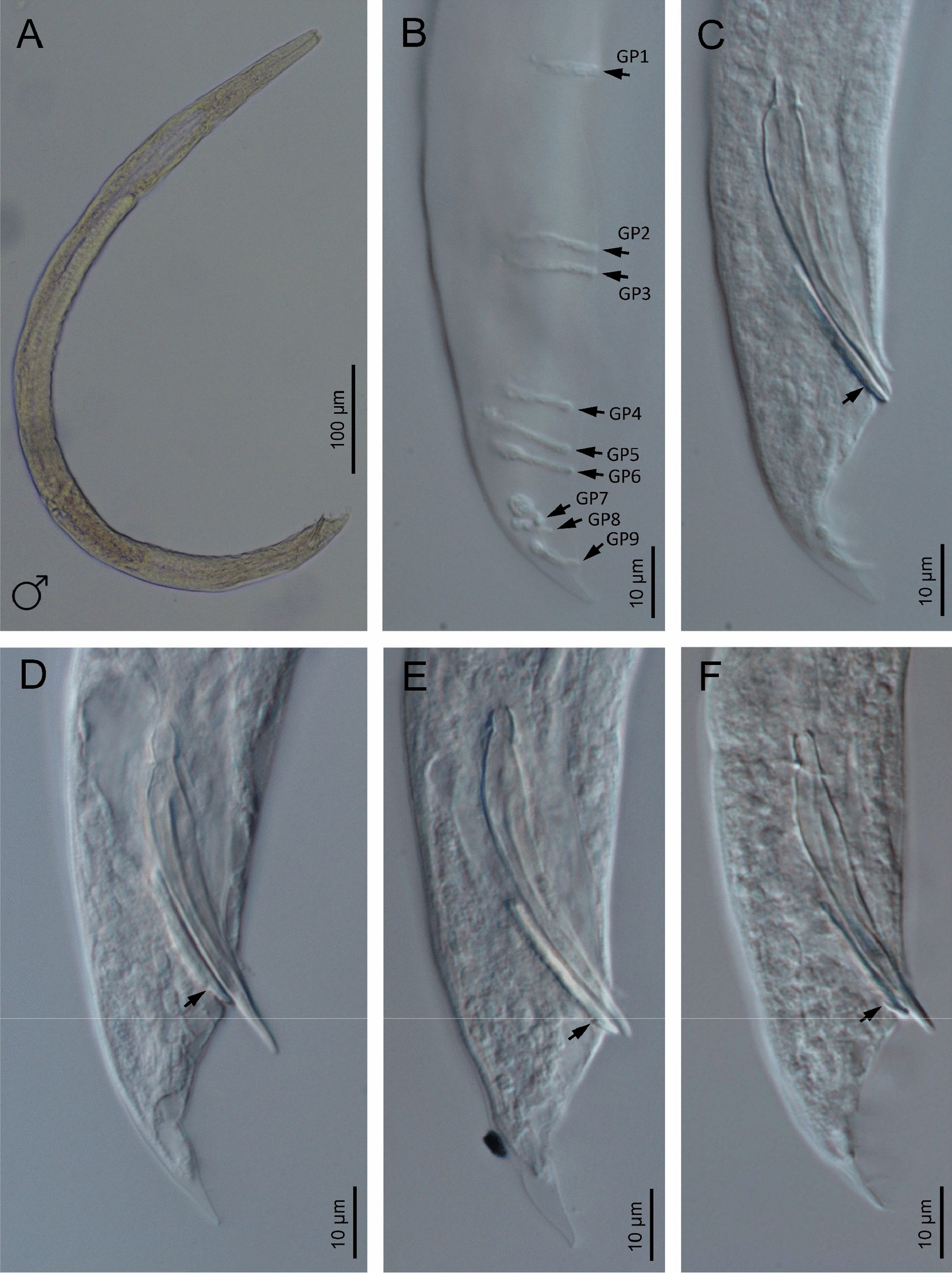
Light microscope (LM) photographs of *H. americana* n. sp. S8 males. **A** Entire body. **B** Posterior end at bursa level (arrows pointing at the genital papillae). **C**–**F** Posterior end at spicules level (arrow pointing at the harpoon-like terminus of the gubernaculum)

**Fig. 4:**
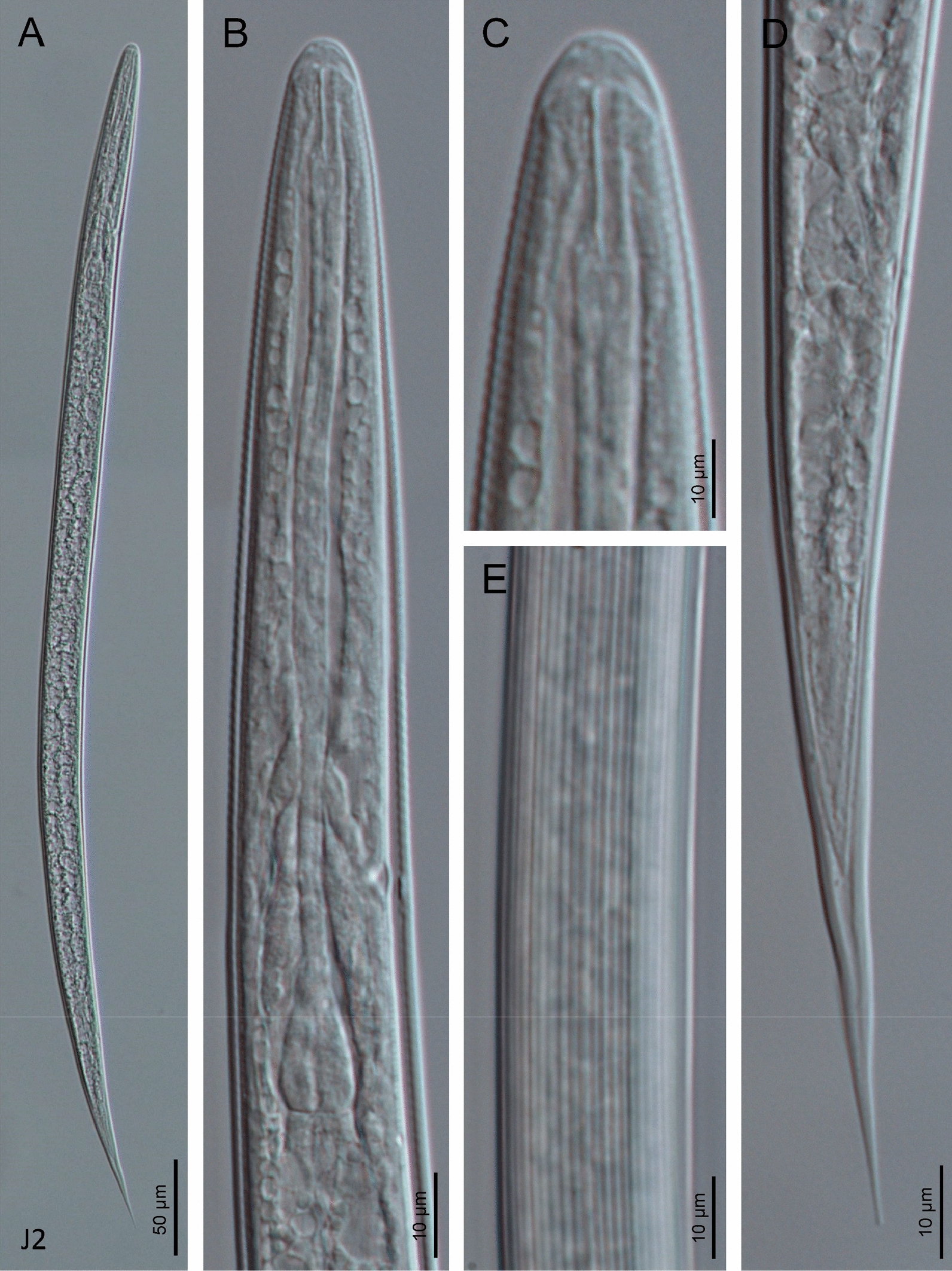
Light microscope (LM) micrographs of *H. americana* n. sp. S8 third-stage (J3) juveniles ensheathed in the J2 cuticle. **A** Entire body. **B** Pharyngeal region. **C** Anterior end. **D** Tail region. **E** Cuticle

**Fig. 5:**
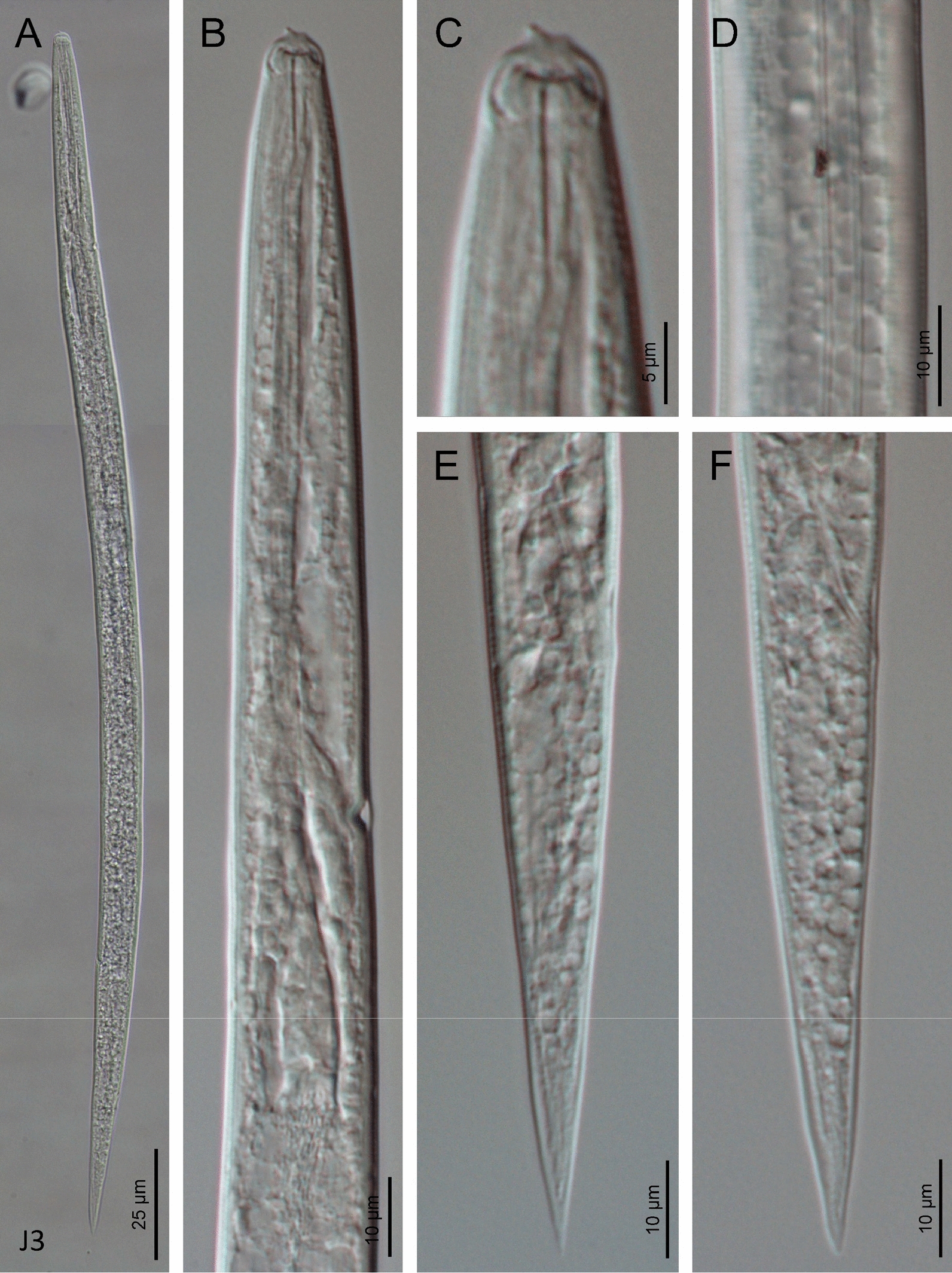
Light microscope (LM) micrographs of *H. americana* n. sp. S8 third-stage (J3) juveniles. **A** Entire body. **B** Pharyngeal region. **C** Anterior end. **D** Cuticle. **E**–**F** Tail region

**Fig. 6:**
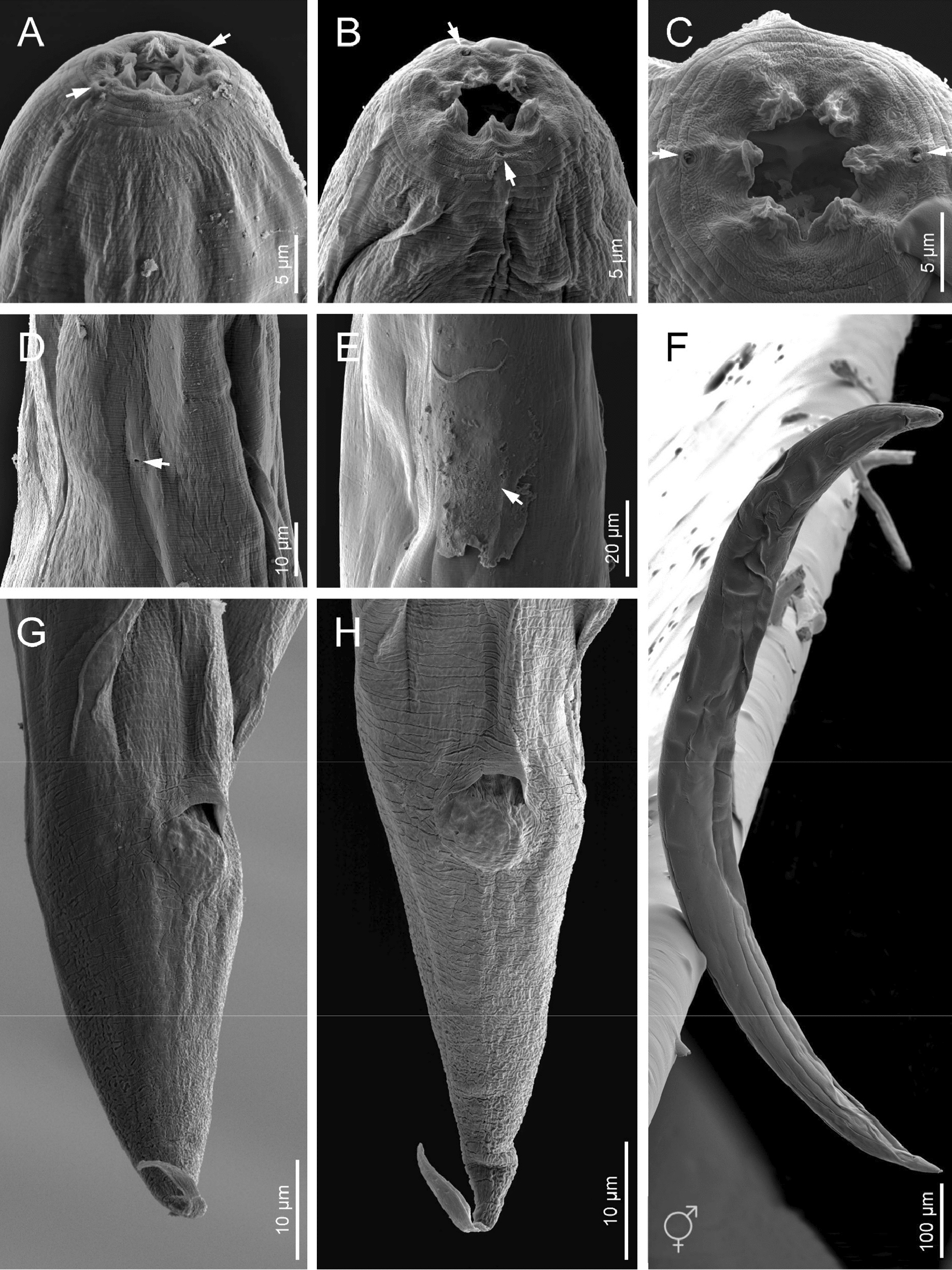
Scanning electron microscope (SEM) photographs of *H. americana* n. sp. S8 hermaphroditic females. **A**–**C** Anterior ends in sub-ventral, lateral, and frontal views, respectively (white arrows pointing at the amphids). **D** Excretory pore (white arrow). **E** Vulva covered by vaginal plug (arrow). **F** Entire body. **G**–**H** Posterior ends in sub-ventral and ventral views, respectively

**Fig. 7:**
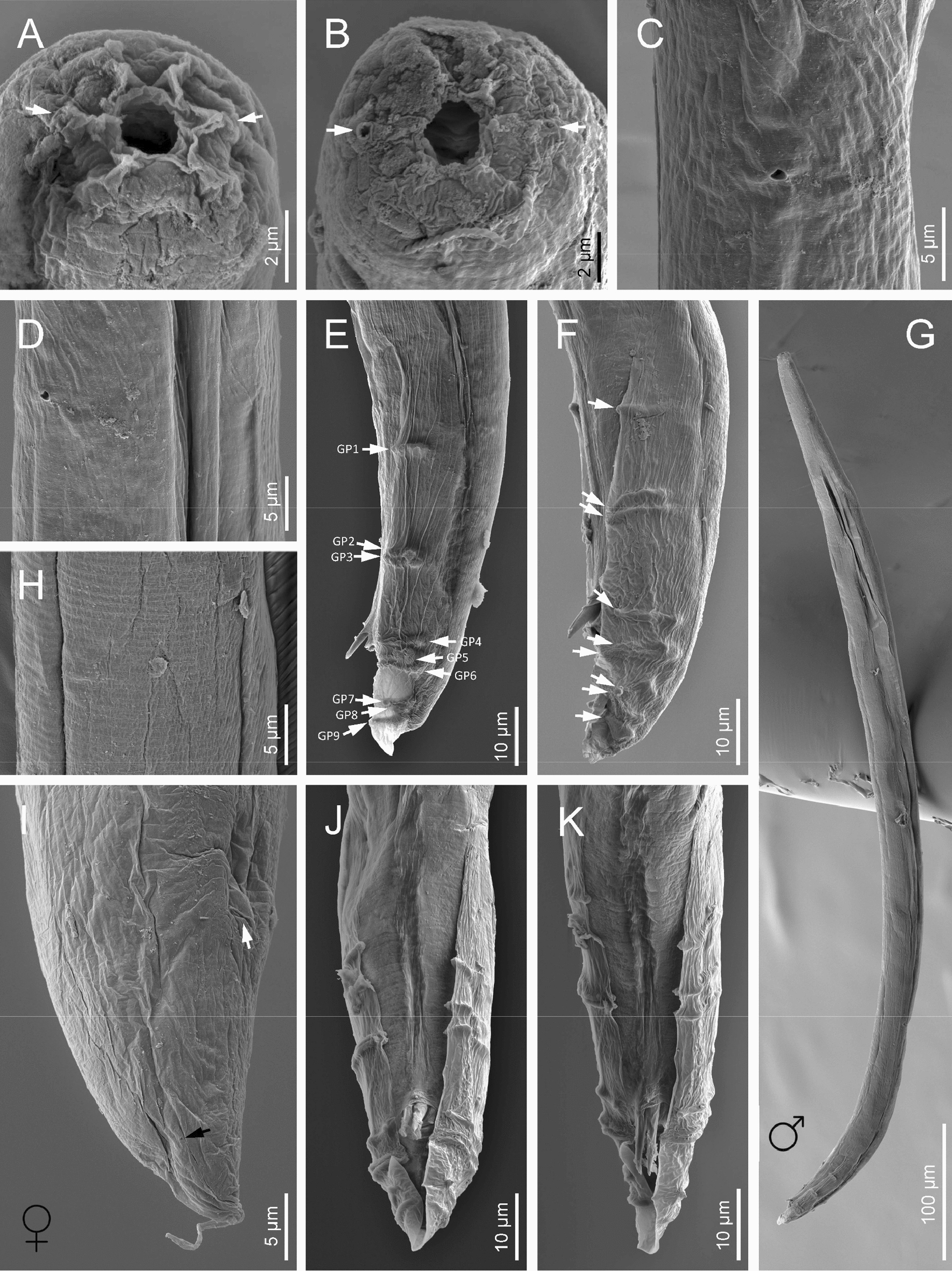
Scanning electron microscope (SEM) photographs of *H. americana* n. sp. S8 dioecious adults. **A**, **B** Anterior ends of a female and a male, respectively, in frontal view, (white arrows pointing at the amphids). **C**, **D** Excretory pores of a male in ventral view and a female in sub-ventral view. **E**, **F** Posterior end of a male adult in lateral view (arrows pointing at the genital papillae). **G** Entire body of a male. **H** Cuticle of a female adult. **I** Tail of a female adult in sub-ventral view (white arrow pointing at the anus, black arrow pointing at the phasmid). **J**, **K** Posterior end of a male adult in ventral view

**Fig. 8:**
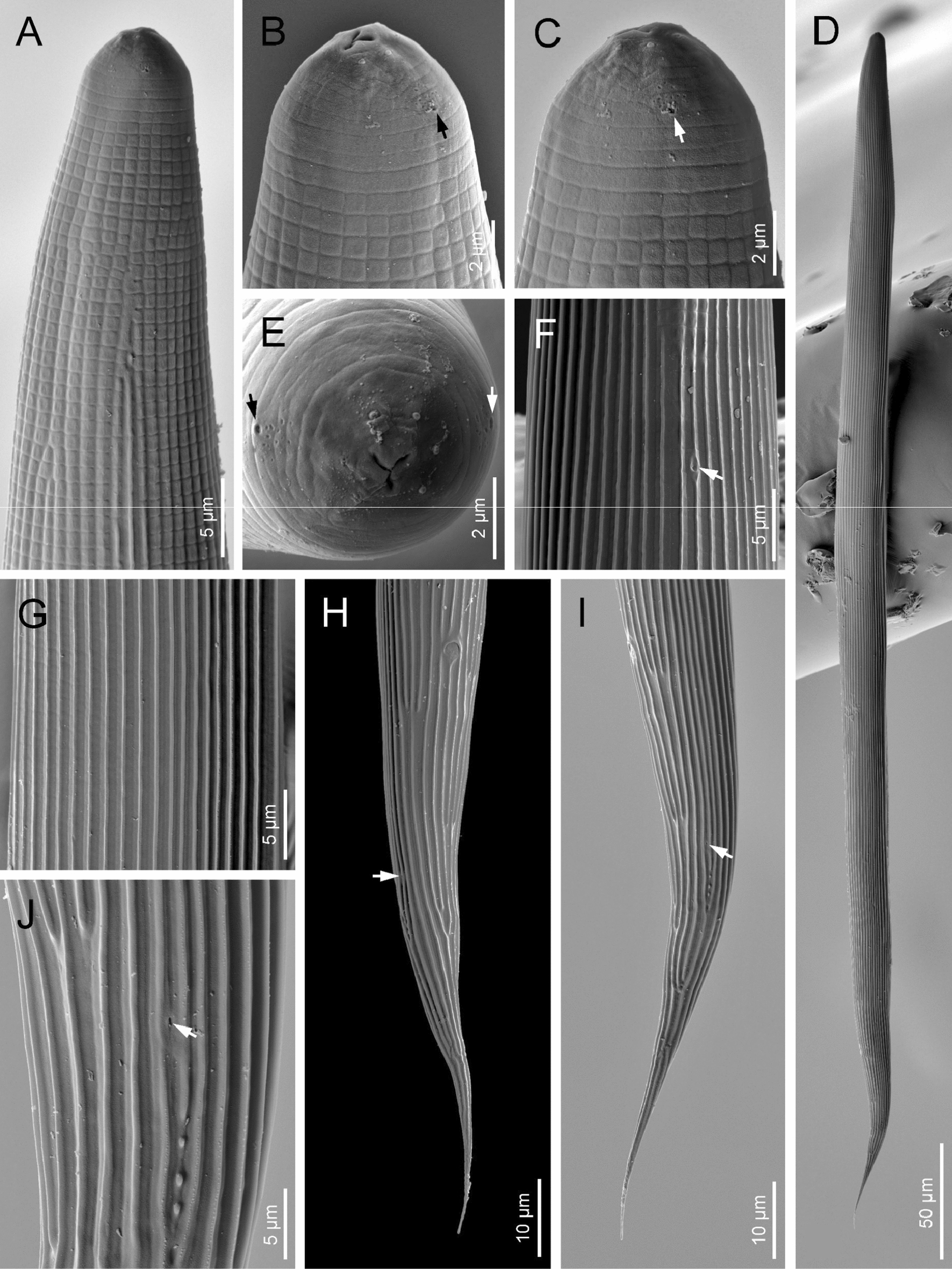
Scanning electron microscope (SEM) photographs of *H. americana* n. sp. S8 third-stage (J3) juveniles ensheathed in the J2 cuticle. **A** Pharyngeal region in lateral view. **B**, **C**, **E** Anterior end in sub-ventral, lateral and frontal views, respectively (arrows pointing at the amphids). **D** Entire body. **F** Excretory pore (arrow). **G** Cuticle at midbody. **H**, **I** Tail in ventral and lateral views, respectively (arrows pointing at the phasmids). **J** Left phasmid (arrow)

**Fig. 9:**
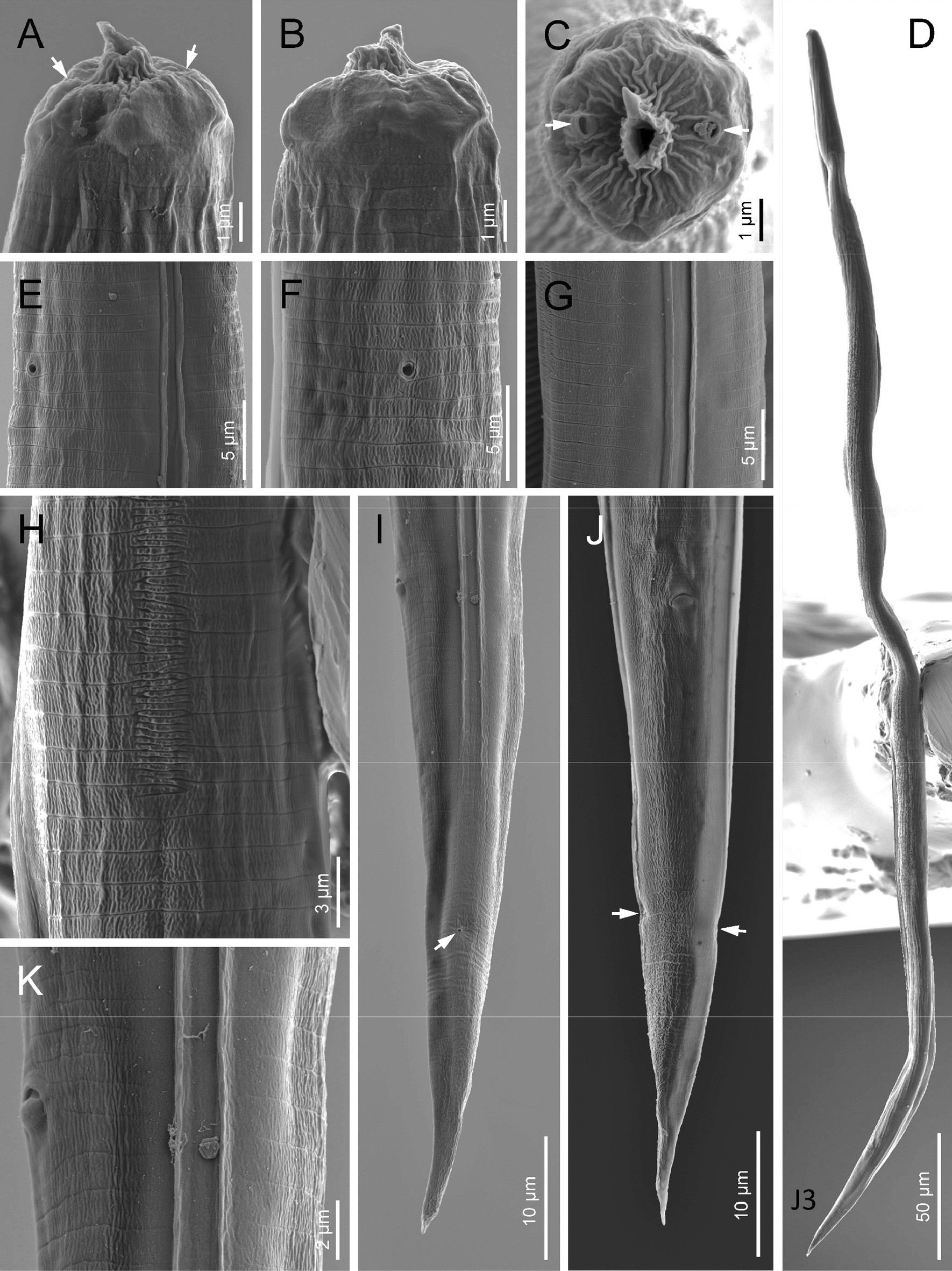
Scanning electron microscope (SEM) photographs of *H. americana* n. sp. S8 third-stage juvenile (J3). **A**–**C** Lip region in ventral, sublateral, and frontal views, respectively (arrows pointing at the amphids). **D** Entire body. **E**, **F** Excretory pore at lateral and ventral views, respectively. **F** Lateral field. **H** Cuticle at anterior end in lateral view. **I**, **J** Posterior end in lateral and ventral views, respectively (arrows pointing at the phasmid). **K** Lateral field at anus level

**Table 1:** Morphometric values of infective juveniles (IJs) and adult generations of *H. americana* n. sp. nematodes

Character	Male		Hermaphrodite paratypes	Female paratypes	Infective juvenile paratypes
	Holotype	Paratype			
*n*	1	15	15	15	15
Body length (L)	878	806.6 ± 71.1 (694–878)	2644.8 ± 460.7 (1918–3190)	1898.4 ± 450.7 (1120–2425)	537 ± 42 (474–617)
*a*	21.4	20.8 ± 1.4 (19.1–22.3)	13.5 ± 0.6 (12.8–14.4)	15.1 ± 0.7 (14.3–16.1)	26.0 ± 2.6 (23.1–30.8)
*b*	8.7	7.6 ± 0.7 (6.5–8.7)	16.8 ± 2.2 (13.0–18.5)	14.20 ± 1.2 (12.4–16.3)	4.9 ± 0.4 (4.4–5.6)
*c*	36.6	32.1 ± 5.1 (26.7–40.2)	42.3 ± 8.1 (29.5–49.3)	31.1 ± 4.4 (28.3–40.0)	6.3 ± 1.1 (5.3–8.4)
*c*'	1.1	1.2 ± 0.2 (0.9–1.4)	2.4 ± 0.3 (2.2–2.8)	2.7 ± 0.4 (2.3–3.1)	8.0 ± 1.2 (6.5–9.4)
*V*	70.5	75.4 ± 15.0 (58–90)	50.0 ± 1.3 (48–51)	48.8 ± 2.7 (47–54)	–
Lip region diameter	12	11.7 ± 0.8 (11–13)	17.2 ± 2.9 (15–22)	15.2 ± 2.2 (11–17)	6.3 ± 0.5 (6–7)
Stoma length	10	10.2 ± 1.5 (9–12)	12.6 ± 1.5 (11–15)	11.1 ± 1.6 (9–13)	12.0 ± 1.4 (11–14)
Corpus (procorpus + median bulb)	58	59.0 ± 1.4 (58–60)	83.3 ± 8.4 (78–93)	81.2 ± 2.9 (76–85)	60.0 ± 5.7 (56–64)
Isthmus	11	13.5 ± 3.5 (11–16)	31.3 ± 6.0 (25–37)	22.3 ± 2.9 (20–25)	17.5 ± 4.9 (14–21)
Bulb length	19	19.5 ± 1.0 (19–21)	37.0 ± 3.6 (34–41)	29.3 ± 1.2 (27–30)	14.6 ± 0.8 (14–16)
Cardia length	-	-	10.3 ± 2.8 (7–13)	7.5 ± 1.3 (6–9)	2.8 ± 0.2 (2.6–3.0)
Nerve ring to anterior end (NR)	64	72.2 ± 5.0 (64–78)	116.0 ± 13.2 (98–134)	103.3 ± 8.2 (91–112)	76.2 ± 9.6 (64–87)
Excretory pore to anterior end (EP)	114	111.0 ± 2.6 (109–114)	162.4 ± 9.2 (148–169)	113.8 ± 16.9 (101–136)	95.0 ± 4.3 (90–101)
Pharynx length	91	95.0 ± 3.6 (91–98)	144.8 ± 11.1 (137–161)	129.0 ± 8.4 (121–140)	98.3 ± 9.5 (91–109)
Neck length (NL)	109	106.7 ± 4.2 (101–113)	154.0 ± 14.7 (148–183)	140.2 ± 11.9 (132–160)	115.8 ± 11.6 (112–138)
Body diameter at neck base	30	28.4 ± 3.7 (24–32)	84.2 ± 5.7 (77–90)	56.0 ± 6.8 (43–62)	18.9 ± 2.6 (16–22)
Body diameter at midbody (BD)	41	38.9 ± 3.9 (33–43)	196.6 ± 32.8 (140–221)	131.7 ± 21.2 (116–169)	20.5 ± 2.3 (17–24)
Body diameter at anus	21	20.7 ± 1.0 (19–22)	27.6 ± 4.2 (23–34)	22.4 ± 2.3 (21–26)	11.1 ± 1.8 (9–14)
Vulva–anterior end	–	–	1317.6 ± 206.0 (976–1536)	977.4 ± 124.3 (874–1194)	–
Rectum	–	–	58.8 ± 11.1 (47–77)	41.7 ± 5.8 (36–47)	–
Tail length (T)	24	25.6 ± 3.9 (18–29)	63.2 ± 9.4 (54–76)	64.3 ± 7.3 (53–73)	86.0 ± 12.5 (64–100)
Tail hyaline length	–	–	18.0 ± 4.4 (13–25)	14.5 ± 2.4 (11–17)	36.7 ± 6.5 (30–50)
Spicule length (SL)	46	40.4 ± 4.8 (36–48)	–	–	–
Gubernaculum length (GL)	20	17.4 ± 3.6 (13–23)	–	–	–
Stoma length/lip region width	0.8	0.9 ± 0.1 (0.7–1.0)	0.7 ± 0.1 (0.7–0.9)	0.7 ± 0.1 (0.6–0.8)	1.9 ± 0.1 (1.8–2.0)
Nerve ring% neck length	63.4	67.5 ± 4.4 (63–73)	76.0 ± 0.8 (75–77)	73.3 ± 1.0 (72–75)	0.7 ± 0.0 (0.6–0.7)
Excretory pore% neck length	112.9	106.2 ± 5.8 (103–113)	104.1 ± 9.4 (91–113)	81.4 ± 6.8 (77–90)	0.8 ± 0.1 (0.7–0.9)
Rectum% ABD	–	–	226.8 ± 26.3 (200–266)	18.5 ± 21.3 (169–224)	–
D% (EP/NL*100)	112.9	106.1 ± 5.8 (102–113)	101.8 ± 9.08 (91–113)	84.09 ± 6.95 (76–90)	80.8 ± 5.7 (74–87)
E% (EP/T*100)	475.0	444.7 ± 28.2 (419–475)	262.4 ± 48.3 (209–313)	182.7 ± 41.0 (153–257)	105.4 ± 21.8 (92–138)
SW% (SP/ABD*100)	219.0	187.3 ± 19.5 (168–219)	–	–	–
GS% (GL/SL*100)	43.5	41.9 ± 4.4 (35–47)	–	–	–
H% (H/T*100)	–	–	28.7 ± 6.3 (20–33)	22.9 ± 1.5 (21–24)	43.1 ± 7.5 (34–55)
Vulva posterior end	–	–	–	–	–
Male reproductive system	619	619.0 ± 136.1 (411–742)	–	–	–
Testis	94	99.0 ± 23.4 (67–124)	0.7 ± 0.1 (0.7–0.9)	–	–
Egg diameter	–		–	50.4 ± 2.2 (47–53)	

All characters are presented in µm (except ratios and percentages) and given as mean ± standard deviation (range)

**Table 2:** Comparative morphometric values of *Heterorhabditis* adult males

Species	L	BD	EP	NR	NL	T	SL	GL	*a*	*b*	*c*	*c*′	SW%	GS%	D%	Country	Reference
*H. amazonensis*	692–826	36–43	96–116	71–88	97–114	29–41	35–45	19–23	18.7^a^	7.7^b^	27.5^b^	1.3^b^	120–187	44–56	95–109	Brazil	Andaló et al. [28]
***H. americana* n. sp.**	**694**–**878**	**33**–**43**	**109**–**114**	**64**–**78**	**101**–**113**	**18**–**29**	**36**–**48**	**13**–**23**	**19**–**22**	**6.5**–**8.7**	**27**–**40**	**0.9**–**1.4**	**168**–**219**	**35**–**47**	**102**–**113**	**USA**	**This study**
*H. atacamensis*	842–1025	42–55	116–149	69–93	99–119	24–36	40–49	17–22	19.7^a^	9.6^b^	29.3^b^	1.5^b^	179–249	38–51	108–126	Chile	Edgington et al. [37]
*H. bacteriophora*	780–960	38–46	114–130	65–81	99–105	22–36	36–44	18–25	20.8^a^	9.1^a^	34.3^a^	1.8^a^	174	50	117	Australia	Poinar [25]
*H. baujardi*	818–970	45–53	71–93	54–77	105–132	28–38	33–45	18–22	16–22	6.4–8.8	24–33	1.5^b^	138–208	44–61	79^b^	Vietnam	Phan et al. [34]
*H. beicherriana*	889–1192	51–73	130–157	81–108	116–143	32–45	40–49	22–27	15–23	7.2–10	22–34	1.3–2.3	153–208	48–59	102–120	China	Li et al. [38]
*H. casmirica*	608–914	24–48	102–120	58–80	100–118	16–32	38–48	18–26	15–24	6.4–8.2	24–35	1.1–1.6	160–252	45–63	99–107	India	Bhat et al. [24]
*H. downesi*	699–876	33–40	86–91	62–78	97–106	29–34	41–47	17–19	26.6^a^	8.8^b^	27.4^b^	1.4^b^	170–220	36–47	90	Ireland	Stock et al. [39]
*H. egyptii*	594–848	31–56	80–97	56–84	96–109	23–34	25–50	16–22	17.1^a^	6.6^b^	19.5^b^	1.5^a^	120–220	40–65	84–91	Egypt	Abd-Elgawad & Ameen [53]
*H. floridensis*	785–294	43–50	104–128	73–90	97–111	29–40	36–46	17–30	19.9^a^	7.9^b^	24.1^b^	1.4^b^	133–209	47–65	112	USA	Nguyen et al. [30]
*H. georgiana*	721–913	43–55	101–145	72–93	100–122	29–41	41–49	20–28	16.5^a^	7.7^b^	26.1^b^	1.4^b^	150–200	51–64	100–122	USA	Nguyen et al. [32]
*H. hambletoni*	510–800	38–60	80–100	80–90	–	–	–	–	–	–	–	–	–	–	–	Brazil	Pereira [42]
*H. indica*	573–788	35–46	109–138	72–85	93–109	24–32	35–48	18–23	17.6^a^	6.7^b^	23.0^b^	1.1^b^	187	49	121	India	Poinar et al. [36]
*H. marelatus*	805–1046	48–56	110–168	61–95	99–123	24–38	41–49	18–22	15.5^a^	7.8^b^	30.0^b^	1.1^b^	196	36–50	113^b^	USA	Liu & Berry [33]
*H. megidis*	800–1100	44–50	139–176	96–112	122–134	35–43	46–54	17–24	18–22	7–9	23–31	1.6^a^	188	43	122	USA	Poinar et al. [35]
*H. mexicana*	614–801	38–47	108–145	61–83	89–108	21–36	30–47	18–32	21.7^a^	6.8^b^	27.6^b^	1.1^b^	130–196	43–70	114–149	Mexico	Nguyen et al. [29]
*H. noenieputensis*	530–775	34–46	75–102	64–75	88–106	21–32	37–49	17–24	14–18	5.6–7.9	21–33	1.1–1.7	202–301	38–56	81–108	SA	Malan et al. [27]
*H. ruandica*	652–863	40–51	61–109	56–74	84–117	21–29	34–50	16–23	15–21	5.8–9.7	23–36	0.6–1.7	150–306	35–57	61–97	Rwanda	Machado et al. [93]
*H. safricana*	777–1009	40–58	104–147	52–61	105–126	27–49	35–54	19–27	20.1^a^	7.9^b^	43.0^b^	1.5^a^	130–259	43–62	92–133	SA	Malan et al. [31]
*H. taysearae*	648–736	38–48	78–120	54–88	85–123	20–29	30–42	12–21	15.1^a^	6.5^b^	14.0^b^	1.3^b^	156	46	88	Egypt	Shamseldean et al. [73]
*H. zacatecana*	811–914	41–56	77–109	60–78	71–108	21–33	38–55	15–25	15–25	7.6–12	26–43	1.2–2.5	170–320	40–60	78–134	Mexico	Machado et al. [93]
*H. zealandica*	848–1044	36–45	130–150	–	110–128	30–41	48–55	19–25	–	–	–	1.7^a^	246	44	118	NZ	Poinar [40]

All measurements are presented in µm (except ratios and percentages)

^a^Data calculated from the drawings provided in the original publication

^b^Data calculated from other measurements provided in the original publication

–Data not provided in the original publication

**Table 3:** Comparative morphometric values of *Heterorhabditis* hermaphrodite females

Species	L	BD	EP	NR	NL	T	*a*	*b*	*c*	*c*′	V	ABD	D%	Country	Reference
*H. amazonensis*	3517–5587	220–316	184–238	128–171	180–225	104–154	–	–	–	2.3^a^	42–47	59–83	103^a^	Brazil	Andaló et al. [28]
***H. americana* n. sp.**	**1918**–**3190**	**140**–**221**	**148**–**169**	**98**–**134**	**148**–**183**	**54**–**76**	**13**–**14**	**13**–**19**	**30**–**49**	**2.2**–**2.8**	**48**–**51**	**23**–**34**	**91**–**113**	**USA**	**This study**
*H. atacamensis*	1791–2904	88–122	165–206	101–132	174–200	72–112	–	–	–	2.7^a^	39–48	30–46	90–114	Chile	Edgington et al. [37]
*H. bacteriophora*	3630–4390	160–180	189–217	121–130	189–205	81–93	–	–	–	–	41–47	40–53	106	Australia	Poinar [25]
*H. baujardi*	3135–4170	180–240	156–192	119–147	186–206	66–114	15–19	16–21	36–50	2.0^a^	43–48	47–63	88^a^	Vietnam	Phan et al. [34]
*H. beicherriana*	3671–5543	198–374	165–297	135–243	192–343	68–130	13–20	13–25	34–62	1.0–2.3	41–49	51–92	76–94	China	Li et al. [38]
*H. casmirica*	2851–4219	140–341	180–211	77–100	174–207	72–114	19–27	16–39	56–84	1.4–2.2	46–57	36–56	94–120	India	Bhat et al. [24]
*H. downesi*	3030–5051	183–291	200–254	175–230	230–244	60–70	–	–	–	1.1^a^	50–55	57–65	117^a^	Ireland	Stock et al. [39]
*H. egyptii*	2100–3100	107–164	154–205	101–147	144–192	83–115	–	–	–	2.7^a^	46–59	33–51	104^a^	Egypt	Abd-Elgawad & Ameen [53]
*H. floridensis*	3731–5865	217–331	211–301	169–271	271–391	84–126	–	–	–	2.5^a^	44–49	42–78	104^a^	USA	Nguyen et al. [30]
*H. georgiana*	3232–4928	157–267	200–277	143–217	132–271	65–96	–	–	–	1.2^a^	44–55	42.6^a^	–	USA	Nguyen et al. [32]
*H. hambletoni*	–	–	–	–	–	–	–	–	–	–	–	–	–	Brazil	Pereira [42]
*H. indica*	2300–3100	107–145	163–187	104–123	163–179	72–110	–	–	–	–	45–50	38–51	–	India	Poinar et al. [36]
*H. marelatus*	3000–4500	161–233	212–287	133–182	190–244	75–101	–	–	–	1.3^a^	45–50	20–28	109^a^	USA	Liu & Berry [33]
*H. megidis*	2400–4900	120–133	193–270	139–178	106–269	95–124	14–24	12–21	23–49	–	45–50	36–86	–	USA	Poinar et al. [35]
*H. mexicana*	2440–4606	135–267	103–201	114–171	168–221	94–170	–	–	–	2.6^a^	30–58	40–46	90^a^	Mexico	Nguyen et al. [29]
*H. noenieputensis*	2987–5498	168–289	152–209	112–152	166–220	79–120	14–23	18–28	37–58	1.7–3.4	39–47	26–56	77–112	S. Africa	Malan et al. [27]
*H. ruandica*	2907–4123	209–274	106–153	78–108	134–159	63–98	12–16	21–27	34–51	1.7–2.6	45–55	29–51	67–103	Rwanda	Machado et al. [93]
*H. safricana*	3373–4073	127–188	210–267	121–163	199–236	64–91	–	–	–	–	43–46	40–54	98–119	SA	Malan et al. [31]
*H. taysearae*	2200–2800	116–170	137–182	83–120	161–200	72–100	–	–	–	–	40–64	41–67	–	Egypt	Shamseldean et al. [73]
*H. zacatecana*	4408–6179	235–385	108–190	96–169	174–231	63–87	13–20	20–34	52–90	1.2–2.4	36–57	34–58	55–95	Mexico	Machado et al. [93]
*H. zealandica*	–	–	–	–	–	–	–	–	–	–	–	–	–	NZ	Poinar [40]

All measurements are presented in µm (except ratios and percentages)

^a^Data calculated from the drawings provided in the original publication

(–) Data not provided in the original publication

**Table 4:** Comparative morphometric values of *Heterorhabditis* adult females

Species	L	BD	EP	NR	NL	T	*a*	*b*	*c*	*c*′	V	ABD	D%	Country	Reference
*H. amazonensis*	1279–2070	70–122	103–126	68–100	119–142	25–38	–	–	–	2.4^a^	46–50	25–38	–	Brazil	Andaló et al. [28]
***H. americana* n. sp.**	**1120–2425**	**116–169**	**101–136**	**97–112**	**132–160**	**53–73**	**14–16**	**12–16**	**28–40**	**2.3–3.1**	**47–54**	**21–26**	**76–90**	**USA**	**This study**
*H. atacamensis*	1754–2628	86–129	154–182	79–119	129–167	80–108	–	–	–	3.8^a^	43–49	24–33	100–113	Chile	Edgington et al. [37]
*H. bacteriophora*	3180–3850	160–220	174–214	93–118	155–183	71–93	21.4^a^	18.8	41.5^a^	3.1^a^	42–53	22–31	114	Australia	Poinar [25]
*H. baujardi*	1335–2130	90–150	104–149	75–122	131–185	68–89	12–16	10–12	19–32	–	46–51	27–41	–	Vietnam	Phan et al. [34]
*H. beicherriana*	1581–3026	125–218	95–165	59–138	105–186	68–105	10–18	10–23	19–34	1.6–2.4	41–49	35–81	88–98	China	Li et al. [38]
*H. casmirica*	1273–1990	73–150	135–157	84–111	132–156	64–83	14–15	10–13	16–31	1.6–2.5	45–52	22–30	99–116	India	Bhat et al. [24]
*H. downesi*	1231–2728	74–131	99–126	117–151	111–155	70–122	–	–	–	2.5^a^	47–60	25–38	–	Ireland	Stock et al. [39]
*H. egyptii*	1050–1420	56–84	69–106	69–94	106–125	56–78	17.5^b^	14.4^b^	22.2^b^	3.1^b^	44–51	19–27	78^b^	Egypt	Abd-Elgawad & Ameen [53]
*H. floridensis*	2054–2548	120–156	110–168	86–122	126–178	69–87	–	–	–	–	44–50	32–42	–	USA	Nguyen et al. [30]
*H. georgiana*	1640–2779	101–188	111–177	96–162	136–219	62–88	–	–	–	1.5^a^	46–53	42^a^	–	USA	Nguyen et al. [32]
*H. hambletoni*	600–1200	70–100	80–90	70–80	–	–	–	–	–	–	50–58^b^	–	–	Brazil	Pereira [42]
*H. indica*	1200–1800	76–113	118–138	88–96	120–139	66–88	–	–	–	–	40–53	22–32	–	India	Poinar et al. [36]
*H. marelatus*	1600–2600	113–177	139–178	79–119	129–164	55–81	–	–	–	1.3^a^	45–50	29–48	110^a^	USA	Liu & Berry [33]
*H. megidis*	1500–2500	95–140	158–206	105–120	155–168	70–101	15–19	10–16	18–32	2.6^a^	47–51	25–38	119^a^	USA	Poinar et al. [35]
*H. mexicana*	1144–2108	65–123	114–148	76–103	121–150	76–106	–	–	–	–	44–51	21–36	–	Mexico	Nguyen et al. [29]
*H. noenieputensis*	1075–1697	76–129	102–125	73–90	115–132	63–75	13–17	9–14	17–24	2.3–3.1	40–53	22–32	83–104	SA	Malan et al. [27]
*H. ruandica*	1131–1608	68–83	92–129	69–97	107–132	62–88	15–20	9.0–14	16–24	1.9–3.6	41–51	18–34	74–104	Rwanda	Machado et al. [93]
*H. safricana*	1679–2937	102–229	151–196	87–139	148–180	55–111	–	–	–	1.3^a^	45–50	25–72	97–120	S. Africa	Malan et al. [31]
*H. taysearae*	830–1400	42–96	120–166	76–109	129–179	62–80	–	–	–	4.0^a^	44–73	19–28	82^a^	Egypt	Shamseldean et al. [73]
*H. zacatecana*	1954–2798	160–228	100–133	71–96	112–148	45–75	11–15	16–21	31–63	1.3–2.0	43–61	31–41	80–111	Mexico	Machado et al. [93]
*H. zealandica*	–	–	–	–	–	–	–	–	–	–	–	–	–	NZ	Poinar [40]

All measurements are presented in µm (except ratios and percentages)

^a^Data calculated from the drawings provided in the original publication

^b^Data calculated from other measurements provided in the original publication

(–) Data not provided in the original publication

**Table 5:** Comparative morphometric values of *Heterorhabditis* infective juveniles

Species	L	BD	EP	NR	NL	T	*a*	*b*	*c*	*c*′	D%	E%	Country	Reference
*H. amazonensis*	567–612	20–24	89–115	76–93	107–132	98–115	24–29	4.4–5.5	5.1–6.1	7.3^a^	83–92	89–109	Brazil	Andaló et al. [28]
***H. americana* n. sp.**	**474–617**	**17**–**24**	**90**–**101**	**64**–**87**	**112**–**138**	**64**–**100**	**23**–**31**	**4.4**–**5.6**	**5.3**–**8.4**	**6.5**–**9.4**	**74**–**87**	**92**–**138**	**USA**	**This study**
*H. atacamensis*	578–666	19–26	101–126	79–101	124–144	94–107	25–31	4.8–5.7	5.7–7.1	5.7^a^	79–94	149–182	Chile	Edgington et al. [37]
*H. bacteriophora*	512–671	18–31	87–110	72–93	100–139	83–112	17–30	4.0–5.1	5.7–7.0	6.0^a^	76–92	103–130	Australia	Poinar [25]
*H. baujardi*	497–595	18–22	91–103	75–86	107–120	83–97	26–30	4.5–5.1	6.0–6.7	7.2^a^	78–88	98–114	Vietnam	Phan et al. [34]
*H. beicherriana*	566–687	21–25	100–122	85–106	118–146	86–111	24–29	4.2–4.9	5.9–6.8	6.0–7.4	80–93	103–121	China	Li et al. [38]
*H. casmirica*	512–599	17–24	98–129	79–94	114–138	85–115	20–25	4.0–5.2	4.7–6.4	5.1–8.0	83–97	93–136	India	Bhat et al. [24]
*H. downesi*	588–692	15–22	96–128	96–105	126–141	62–74	29–42	4.4–5.3	8.5–10.5	4.4^a^	76–96	160–180	Ireland	Stock et al. [39]
*H. egyptii*	484–515	18–23	81–94	78–100	100–119	53–75	20–27	4.2–5.2	6.8–9.1	6.9^a^	74–82	100–170	Egypt	Abd-Elgawad & Ameen [53]
*H. floridensis*	554–609	19–23	101–122	68–107	123–142	91–113	25–32	3.9–4.9	5.3–6.6	7.2^a^	71–90	95–134	USA	Nguyen et al. [30]
*H. georgiana*	547–651	17–26	97–113	74–94	110–139	86–108	23–34	4.1–5.3	5.5–6.9	6.8^a^	70–93	106	USA	Nguyen et al. [32]
*H. hambletoni*	–	–	–	–	–	–	–	–	–	–	–	–	Brazil	Pereira [42]
*H. indica*	479–573	19–22	88–107	72–85	109–123	93–109	25–27	4.3–4.8	4.5–5.6	–	79–90	83–103	India	Poinar et al. [36]
*H. marelatus*	588–700	24–32	81–113	83–113	121–139	99–117	21–29	4.7–5.4	5.5–6.6	3.0^a^	60–86	89–110	USA	Liu & Berry [33]
*H. megidis*	736–800	27–32	123–142	104–115	147–160	112–128	23–38	4.6–5.9	6.1–6.9	6.3^a^	81–91	103–120	USA	Poinar et al. [35]
*H. mexicana*	530–620	20–24	83–109	74–88	104–142	91–106	24–28	4.2–5.1	5.5–6.3	8.3^a^	72–86	87–111	Mexico	Nguyen et al. [29]
*H. noenieputensis*	484–578	21–25	88–105	69–96	79–115	78–95	21–27	4.3–5.2	5.5–6.8	3.4–4.3	81–95	99–125	S. Africa	Malan et al. [27]
*H. ruandica*	496–591	18–27	70–89	52–64	75–102	49–64	20–29	5.1–6.6	7.6–8.6	3.4–5.8	65–98	99–157	Rwanda	Machado et al. [93]
*H. safricana*	550–676	19–23	103–122	86–101	125–141	86–108	25–32	3.9–4.9	5.4–7.5	8.7^a^	80–90	99–133	SA	Malan et al. [31]
*H. taysearae*	332–499	17–23	74–113	58–87	96–130	44–70	18–27	3.4–4.2	6.5–8.7	3.7^a^	71–96	110–230	Egypt	Shamseldean et al. [73]
*H. zacatecana*	493–578	23–27	72–99	69–72	78–99	52–63	19–24	5.3–7.2	8.2–10	4.3–6.7	72–122	128–184	Mexico	Machado et al. [93]
*H. zealandica*	570–740	22–30	94–123	90–107	135–147	87–119	25	4.9	6.7	–	73–92	103–109	NZ	Poinar [40]

All measurements are presented in µm (except ratios and percentages)

^a^Data calculated from the drawings provided in the original publication

(–) Data not provided in the original publication

### Hermaphroditic females

Hermaphroditic female C-shaped after heat relaxation, body robust, always with many juveniles inside, in some specimens few eggs visible. Cuticle smooth under light microscope, about 1 µm thick. Anterior end tapering anteriorly, labial region with six prominent lips, each with a terminal labial papilla. Cephalic papillae not visible under LM. Pore-like amphidial apertures. Rhabditoid stoma, measuring 0.7–0.9 times the width of the lip region. Stoma with short cheilostom, with barely visible, refringent, and rounded cheilorhabdia. Gymnostom well-developed, with refringent bar-like rhabdia, and funnel-shaped stegostom surrounded by the pharyngeal collar, bearing minute rhabdia. Pharynx with slightly swollen metacorpus, subcylindrical procorpus, robust isthmus, and weakly developed, spheroid basal bulb with inconspicuous valves. Nerve ring at 75–77% of neck length, surrounding the isthmus. Excretory pore at 91–113% of neck length, at basal bulb level. Cardia conoid. Reproductive system didelphic-amphidelphic. Ovaries reflexed and well-developed. Oviducts poorly differentiated. Uteri with numerous embryonated eggs. Vagina short. Vulva a transverse slit, with smooth top and prominent lips, located on a slightly protruding area, close to midbody. Rectum slender, about twice the anal body diameter. Anal region swelling posteriorly. Tail conoid with pointed terminus, lacking mucro. Phasmids inconspicuous.

### Amphimictic females

General morphology similar to hermaphroditic females. Body arcuate, tapering towards the anterior end. Labial papillae more acute and prominent than the papillae of hermaphrodites. Reproductive system didelphic-amphidelphic. Well-developed ovaries, reflexed. Oviducts and uteri hardly visible. Very short vagina, and vulva small having transverse slit opening. Rectum shorter than hermaphroditic female rectum, measuring about 1.7–2.2 times the diameter of the anal body. Prominent anal lips. Tail conoid with acute tip, lacking mucro. Phasmids not visible.

### Males

Body curved ventrally (open C-shaped), sometimes straight after heat relaxation. Anterior end truncate. Lip region with six narrowly separated lips, having six conoid liplets at oral margin. Liplet tips with six labial papillae. Lip region with four cephalic papillae, located at the base of the dorsal and ventral lips. Pore-like amphidial aperture, posterior to the lateral lips. Stoma 0.7–1.0 times the width of the lip region, with short cheilostom. Stoma with refringent rounded cheilorhabdia, barely visible. Short gymnostom with refringent bar-like rhabdia, and long, funnel-shaped stegostom surrounded by the pharyngeal collar, bearing small rhabdia. Pharynx with slightly swollen metacorpus, subcylindrical procorpus, isthmus slightly narrower than metacorpus, robust, and basal bulb spheroid and poorly developed, with poorly developed valvular apparatus. Nerve ring surrounding isthmus, located at 63–73% of neck length. Excretory pore at bulb level, located at 103–113% of neck length. Cardia conoid, protruding into intestine. Intestine poorly differentiated, although with narrower walls at anterior end. Reproductive system monorchic, with testis reflexed anteriorly. Well-developed vas deferens. Spicules well-developed, separate, with small almost quadrangular manubrium, calamus developed, and robust lamina with acute tip, prominent dorsal hump, in some specimens, hump not developed. Velum poorly developed ventrally. Gubernaculum robust, straight or slightly curved ventrally, with a size 35–47% of spicule length, with serrated dorsal margin and harpoon-like tip. Tail ventrally curved posteriorly, flanked by the bursa, conoid, with acute tip. Bursa peloderan bearing nine pairs of bursal papillae. The genital papillae arrangement is 1+2/3+3: three precloacal genital papillae, GP1 located 11–22 µm anterior to GP2 and GP3; GP2 and GP3 are equal in length; GP4–9 are post-cloacal; GP4–6 form a group located posterior to cloaca; GP4 is shorter than GP5 and GP6; GP5 and GP6 are of equal length; GP7–9 form a group close to the tail end; GP7 is slightly curved outward; GP8 is shorter than GP7 and GP9.

### Infective sheathed juveniles (J3 stage covered by the J2 stage cuticle)

Body straight after heat relaxation. Second-stage cuticle present. Cuticle with longitudinal ridges, except at the anterior part of body, posterior to the lip region, with tessellate pattern. Lateral fields not differentiated from the cuticular ridges. Lips not differentiated, with six labial papillae. Cephalic papillae not visible. Pore-like amphidial aperture, showing cuticular dimple-like structures at anterior part. Oral opening triradiate, closed. Stoma tubular, about twice the lip region width. Nerve ring at 60–70% of neck length, surrounding the isthmus. Excretory pore located 70–90% of neck length, at basal bulb level. Well-visible hemizonid. Pharynx slender, with corpus subcylindrical, isthmus narrower and basal bulb pyriform without developed valves. Cardia conoid, surrounded by the intestinal tissue. Bacterial pouch not visible. Rectum narrow, about 1.5 times the anal body diameter. Anus poorly developed. Tail conoid-elongate with sharp terminus. Tail without mucro. Terminal hyaline part 34–55% of tail length. Phasmids very small, located at middle length of tail.

### Infective non-sheathed juveniles (J3 stage)

Body straight to slightly curved ventrally after heat relaxation. Cuticle with transverse striae (annuli). Lateral fields with two ridges. Rounded lip region, lacking differentiated lips. Labial and cephalic papillae not visible. Oral opening closed, rounded, with a small dorsal tooth. Amphidial apertures oval. Stoma tubular, measuring 1.8 to 2.0 times the width of the lip region. Pharynx, nerve ring and excretory pore located at a similar position. Well-developed hemizonid. Cardia conoid, surrounded by intestinal tissue. Rectum narrow and poorly visible. Anus closed. Tail conoid with acute tip, and without mucro. Terminal hyaline part present, very short. Phasmids very small, located at posterior part of tail.

## Diagnosis of *Heterorhabditis americana* n. sp.

*Heterorhabditis americana* n. sp. is characterized by a distinct combination of morphological and morphometric characters in males, hermaphroditic females, and IJs (Table 1). In males, the key diagnostic characteristics include a truncated lip region, tail lengths ranging from 18–29 µm, and spicule lengths between 36–48 µm. The ratio of spicule length relative to anal body diameter (SW%) ranges from 168–219, and the ratio of gubernaculum to spicule length (GS%) ranges between 35–47. Males have nine pairs of genital papillae, with three pairs present in the terminal group of the bursa. Hermaphroditic females have pore-like amphids, prominent lips with unique patterns, and non-prominent anal lips. The IJs are characterized by a body length of 0.47–0.57 mm, an excretory pore situated 90–97 µm from the anterior end, a neck length of 90–124 µm, a tail length of 64–100 µm, and a lateral field with 11 ridges (Table 1).

## Morphological relationships of *Heterorhabditis americana* n. sp. with closely related species

Morphologically, *H. americana* n. sp. is similar to *H. bacteriophora*, *H. beicherriana*, *H. casmirica*, *H. egyptii*, *H. georgiana*, *H. ruandica*, and *H. zacatecana*. Various morphological and morphometric traits differ between* H. americana* n. sp. and their closely related species (Tables 2, 3, 4, 5). Males of *H. americana* n. sp. differ from the males of *H. bacteriophora* in the distance from the excretory pore to the anterior end (109–114 vs. 114–130 μm), tail length (18–29 vs. 28–38 µm), and GS% (35–47% vs. 50%). Compared to *H. beicherriana* males, differences include spicule manubrium shape (quadrangular vs. oblong), body size (0.69–0.88 vs. 0.89–1.19 mm), body diameter (33–43 vs. 51–73 μm), excretory pore to anterior end distance (109–114 vs. 130–157 μm), nerve ring to anterior end distance (64–78 vs. 81–108 μm), neck length (101–113 vs. 116–143 μm), tail length (18–29 vs. 32–45 μm), gubernaculum length (13–20 vs. 22–27 µm), and the GS% (35–47 vs. 48–59). Differences with *H. casmirica* males include spicule manubrium shape (rectangular with scarcely refringent walls vs. rectangular with strongly refringent walls), GP1 positioning (more anterior vs. at spicule level), and a lower GS% (35–47% vs. 45–63%). In contrast to *H. egyptii*, the new species has a longer excretory pore to anterior end distance (109–114 vs. 80–97 μm), and higher D% values (111–125 vs. 84–91). Differences with *H. georgiana* include excretory pore position (at the bulb level vs. posterior to the basal bulb), nerve ring to anterior end distance (64–78 vs. 72–93 μm), tail length (18–29 vs. 29–41 µm), mid-body diameter (36–43 vs. 43–55 μm), gubernaculum length (13–20 v 20–28 µm) and GS% (35–47% vs. 51–64%). Differences with *H. ruandica* males include the spicule manubrium shape (well developed, quadrangular with strongly refringent walls vs. poorly developed, triangular, and not refringent), gubernaculum manubrium shape (harpoon-like vs. straight tip), mid-body diameter (33–43 vs. 40–51 µm), excretory pore to anterior end distance (109–114 vs. 61–109 µm) and E% (111–125 vs. 61–97). Lastly, compared to *H. zacatecana*, differences include a quadrangular spicule manubrium with strongly refringent walls (vs. rounded and not refringent), angular anterior end of the spicule manubrium (vs. rounded), hook-like gubernaculum manubrium (vs. slightly curved), longer distance from nerve ring to anterior end (109–114 vs. 77–109 µm), and longer neck length (101–113 vs. 71–108 µm (Table 2).

In the case of hermaphroditic females, *H. americana* n. sp. differs from *H. bacteriophora* in body length (1.92–3.19 vs. 3.63–4.39 mm), excretory pore to anterior end distance (148–169 vs. 189–217 μm), neck length (148–183 vs. 189–205 μm), tail length (54–76 vs. 81–93 µm), anal body diameter (23–29 vs. 40–53 μm), V% value (48–51 vs. 41–47), and D% value (111–125 vs. 106) values. Compared to *H. beicherriana*, differences include anterior end to excretory pore distance (148–169 vs. 165–297 μm), anterior end to nerve ring distance (113–134 vs. 135–243 μm), neck length (148–183 vs. 192–343 μm), tail length (54–76 vs. 68–130 μm), anal body diameter (23–29 vs. 51–92 μm), V% value (48–51 vs. 41–47), and D% value (111–125 vs. 76–94) values. With *H. casmirica,* key differences include excretory pore position (at bulb level vs. more posterior), tail length (72–114 vs. 54–76 μm), and demanian ratios. Differences with *H. egyptii*, include tail length (54–76 vs. 83–115 μm) and anal body diameter (23–29 vs. 33–51 μm). Compared to *H. georgiana*, they differ in body size (1.92–3.19 vs. 3.23–4.93 mm), nerve ring to anterior end distance (113–134 vs. 143–217 μm), distance from anterior end to excretory pore (148–169 vs. 200–277 μm), and anal body diameter (23–29 vs. 42.6 µm). Compared to *H. ruandica*, differences include the distance from the excretory pore to the anterior end (148–169 vs. 106–153 µm), distance from the anterior end to the nerve ring (113–134 vs. 78–108 µm), anal body diameter (23–29 vs. 29–51 µm), *b* ratio (13–18 vs. 21–27), and E% value (111–125 vs. 67–103). Compared to *H. zacatecana*, *H. americana* n. sp. has a shorter body length (1.92–3.19 vs. 4.41–6.18 mm), shorter maximum body diameter (140–221 vs. 235–385 µm), shorter neck length (148–183 vs. 174–231 µm), shorter anal body diameter (23–29 vs. 34–58 μm), lower *b* ratio (13–18 vs. 20–34), lower *c* ratio (30–49 vs. 52–90), and higher D% value (111–125 vs. 55–95) (Table 3).

In the case of amphimictic females, *H. americana* n. sp. differs from *H. bacteriophora* in body length (1.72–2.43 vs. 3.18–3.85 mm), excretory pore to anterior end distance (101–136 vs. 174–214 μm), neck length (132–160 vs. 155–183 μm), tail length (53–73 vs. 71–93 μm), *a* ratio (14–16 vs. 21.4), *b* ratio (12–16 vs. 18.8), and D% (84–102 vs. 114). Compared to *H. beicherriana*, the new species differs in the anal body diameter (21–26 vs. 35–81 μm) and c′ ratio (2.3–3.1 vs. 1.6–2.4). Compared to *H. georgiana*, differences include the anal body diameter (21–26 vs. 42 μm) and c′ ratio (2.3–3.1 vs. 1.5). Compared to *H. casmirica*, the main differences are the excretory pore position (at bulb level vs. more posterior), c ratio (28–40 vs. 16–31), and D% value (76–90 vs. 99–116) values. Compared to *H. egyptii*, key differences include the body diameter (116–169 vs. 56–84 μm), distance from excretory pore to the anterior end (101–136 vs. 69–106 μm), distance from nerve ring to the anterior end (97–112 vs. 69–94 μm), neck length (132–160 vs. 106–125 μm), and D% value (84–102 vs. 78). Compared to *H. ruandica*, the new species can be distinguished by differences in the mid-body diameter (116–169 vs. 68–83 µm), nerve ring to anterior end distance (97–112 vs. 69–97 µm), neck length (132–160 vs. 107–132 µm), and *c* ratio (28–40 vs. 16–24 µm). Differences with *H. zacatecana* are in the mid-body diameter (116–169 vs. 160–228 µm), nerve ring to anterior end distance (97–112 vs. 71–96 µm), anal body diameter (21–26 vs. 31–41 µm), and *b* ratio (12–16 vs. 16–21 µm) (Table 4).

*Heterorhabditis americana* n. sp. IJs can be distinguished from *H. bacteriophora* IJs by a higher c′ ratio (6.5–9.4 vs. 6.0), lack of a visible bacterial sac (invisible in *H. americana* vs. visible in the ventricular part of the intestine in *H. bacteriophora*), and very small phasmids at the posterior tail compared to inconspicuous phasmids in *H. bacteriophora*. Compared to *H. beicherriana*, *H. americana* differs in the shape of amphidial apertures (oval vs. inconspicuous), the bacterial sac (invisible vs. visible), and size of posterior tail phasmids (very small vs. inconspicuous), and the excretory pore to anterior end distance (90–101 vs. 100–122 μm), and the nerve ring to anterior end distance (64–87 vs. 85–106 μm). Compared to *H. casmirica*, IJs of *H. americana* n. sp. can be differentiated by the position of excretory pore, which is located at basal bulb level in *H. americana* sp. n. rather than at the isthmus level. Compared to *H. georgiana*, the IJs of *H. americana* n. sp. lack a visible bacterial sac near the cardia and have very small phasmids at the posterior part of the tail (vs. inconspicuous ones). Differences with *H. egyptii* include a longer tail (64–100 vs. 53–75 µm). Additionally, *H. americana* n. sp. IJs can be differentiated from *H. ruandica* by a longer anterior end to excretory pore distance (90–97 vs. 70–89 µm), greater tail length (64–100 vs. 49–64 µm), and the presence of a smaller cephalic tooth (small vs. large). The new species differs from to *H. zacatecana* by longer tail (64–100 vs. 52–63 μm), and higher *a* ratio (24–29 vs. 19–24). Further morphological differences between males, hermaphroditic females, amphimictic females, and IJs of *H. americana* n. sp. compared to other *Heterorhabditis* species, are outlined in Tables 2, 3, 4, 5.

## Male morphology

**Fig. 10:**
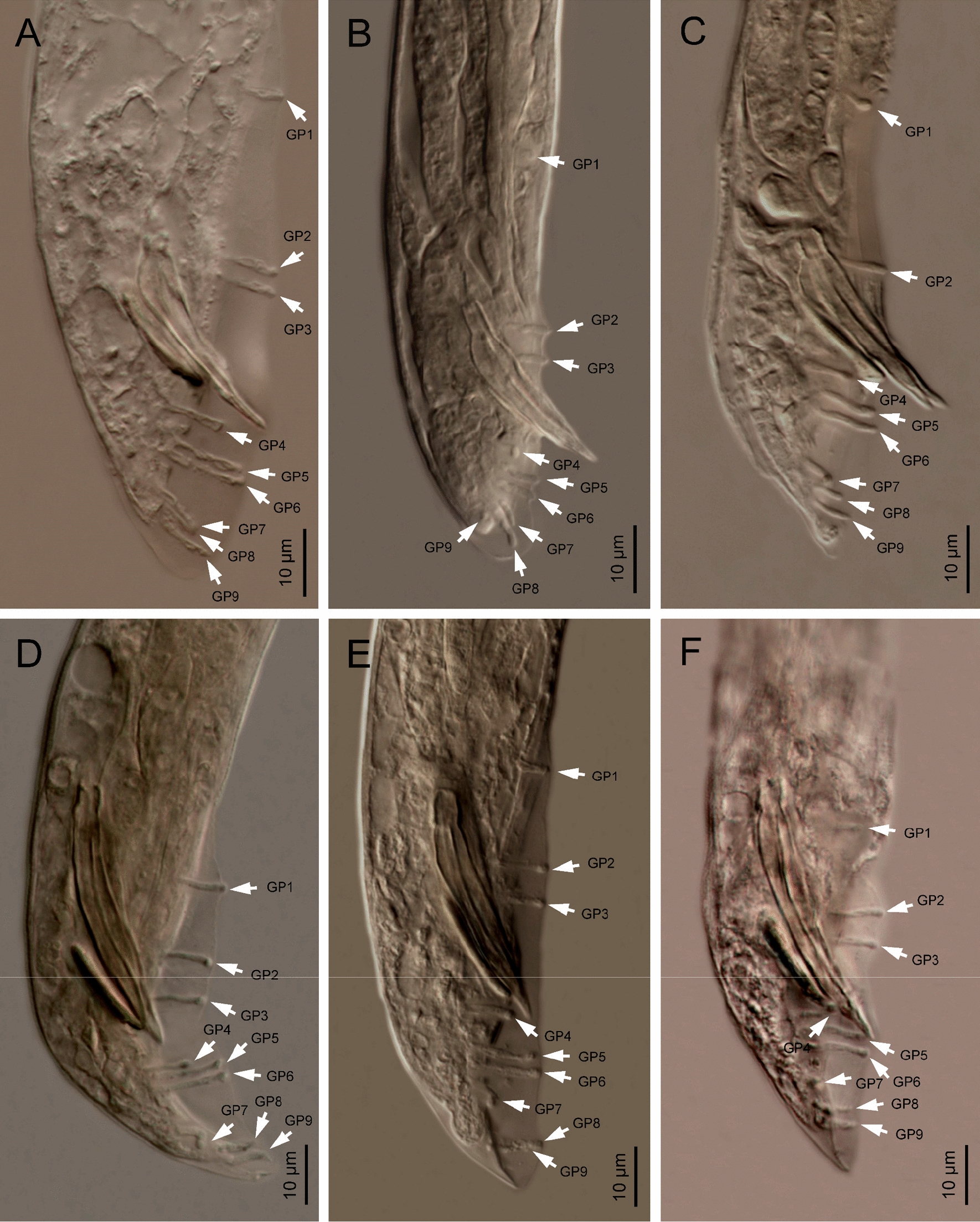
Male tails of *Heterorhabditis beicherriana* and *H. georgiana*. **A** *H. georgiana* (population Kesha, topotype). **B**, **C** *H. georgiana* (population Hbb). **D**–**F** *H. beicherriana* (population M6)

Since *H. americana* n. sp. is morphologically very similar to *H. beicherriana* and *H. georgiana*, the males of three additional isolates, *H. beicherriana* M6, *H. georgiana* (Kesha, topotype), and *H. georgiana* Hbb, were studied. *Heterorhabditis americana* n. sp., *H. beicherriana*, and *H. georgiana* differ particularly in the arrangement of the bursal papillae and the morphology of the spicules (Fig. 10). With respect to the bursal papillae, *H. americana* n. sp. and *H. georgiana* have similar distance between GP1 and GP2–GP3, while *H. beicherriana* has shorter distance between these papillae. Regarding the spicules, all the three species possess manubria of similar shape (rectangular, anteriorly open, and posteriorly thickened outward). However, the lamina lacks a dorsal hump in both *H. americana* and *H. beicherriana*, whereas *H. georgiana* has as a well-developed dorsal hump (Fig. 10).

## Cross-hybridization experiments

No progeny was observed when *H. americana* n. sp. S8 and *H. americana* n. sp. S10 were paired interspecifically with the following nematode isolates: *H. bacteriophora* (Brecon, type), *H. beicherriana* (Cherry, type), *H. casmirica* (HM, type), *H. georgiana* (Kesha, type), *H. ruandica* (Rw14_N-C4a, type), or *H. zacatecana* (MEX-39, type). In contrast, progeny was observed when these isolates were paired intraspecifically. These results show that *H. americana* n. sp. is reproductively isolated from the other *Heterorhabditis* species evaluated in this study. This reproductive isolation supports the classification of *H. americana* n. sp. as a distinct and novel species.

## Nematode molecular characterization and phylogenetic relationships

**Fig. 11:**
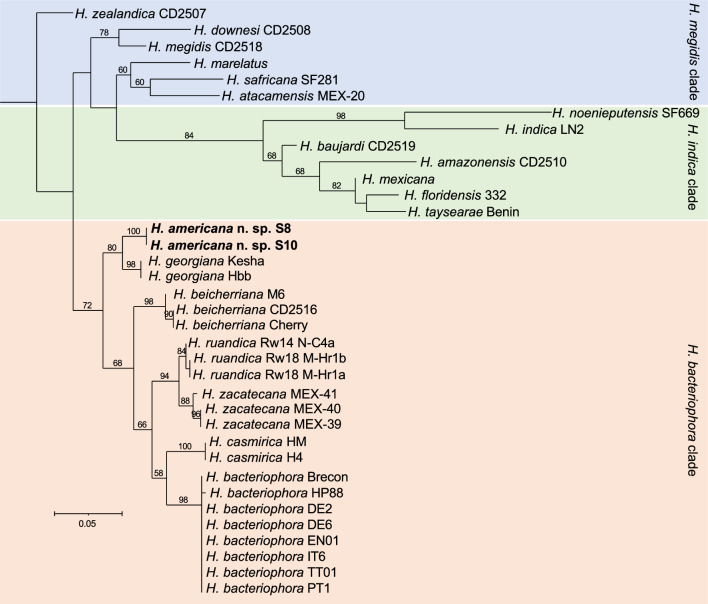
Maximum-likelihood phylogenetic tree reconstructed from the sequences of the mitochondrial cytochrome c oxidase I (*cox-1*) gene. Phylogenetic analyses included 342 nucleotide positions, flanked by primers HCF and HCR. Numbers at nodes represent bootstrap values based on 100 replications. Bars represent average nucleotide substitutions per sequence position. Accession numbers of the nucleotide sequences used for the reconstructions are presented in Table S2

**Fig. 12:**
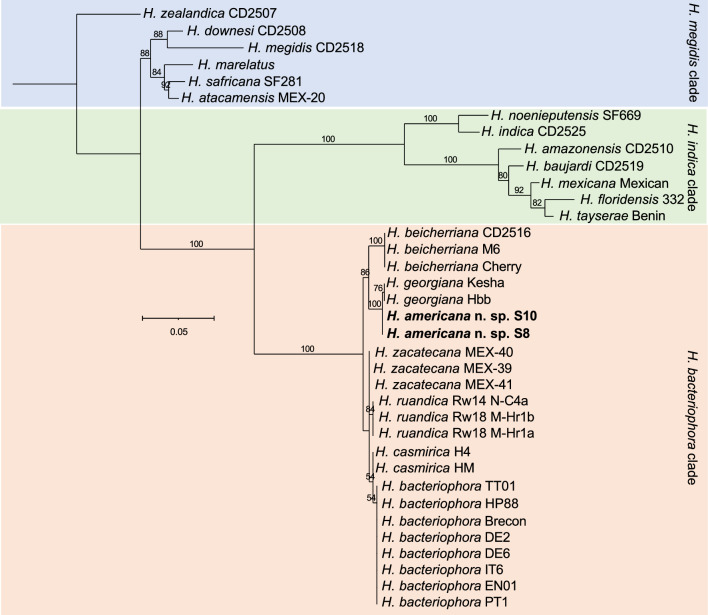
Maximum-likelihood phylogenetic tree reconstructed from sequences of the internal transcribed spacer (ITS) region of the rRNA gene. Phylogenetic analyses included 796 nucleotide positions, flanked by primers TW81 and AB28. Numbers at nodes represent bootstrap values based on 100 replications. Bars represent average nucleotide substitutions per sequence position. Accession numbers of the nucleotide sequences used for the analyses are presented in Table S2

Phylogenetic reconstructions based on mitochondrial cytochrome oxidase subunit 1 (*cox-1*) gene sequences and the ITS region of the rRNA gene show that *H. americana* n. sp. is closely related to *H. georgiana* and belongs to the “*Bacteriophora*” clade (Figs. 11 and 12). This clade includes *H. bacteriophora*, *H. beicherriana*, *H. casmirica, H. georgiana*, *H. ruandica*, and *H. zacatecana* (Figs. 11 and 12). The sequences of the D2–D3 expansion segments of the 28S rRNA gene, however, are of limited phylogenetic value, as closely related species are not phylogenetically resolved using this gene marker (Fig. S1). Sequence similarity scores further support a closer phylogenetic relationship between* H. americana* n. sp. and *H. georgiana* (Figs. S2–S7). The *cox-1* gene sequences of these two species are 96.7% identical, differing by 11 nucleotides. The ITS sequences are 99.8% identical, differing by one nucleotide, and the D2–D3 sequences show no nucleotide differences (Figs. S2–S7). Comparatively lower sequence similarity scores were observed between *H. americana* n. sp. and all the other species of the “*Bacteriophora*” clade (Figs. S2–S7). No intra-individual variability was detected in any of the markers analyzed in this study.

## Symbiotic relationships

**Fig. 13:**
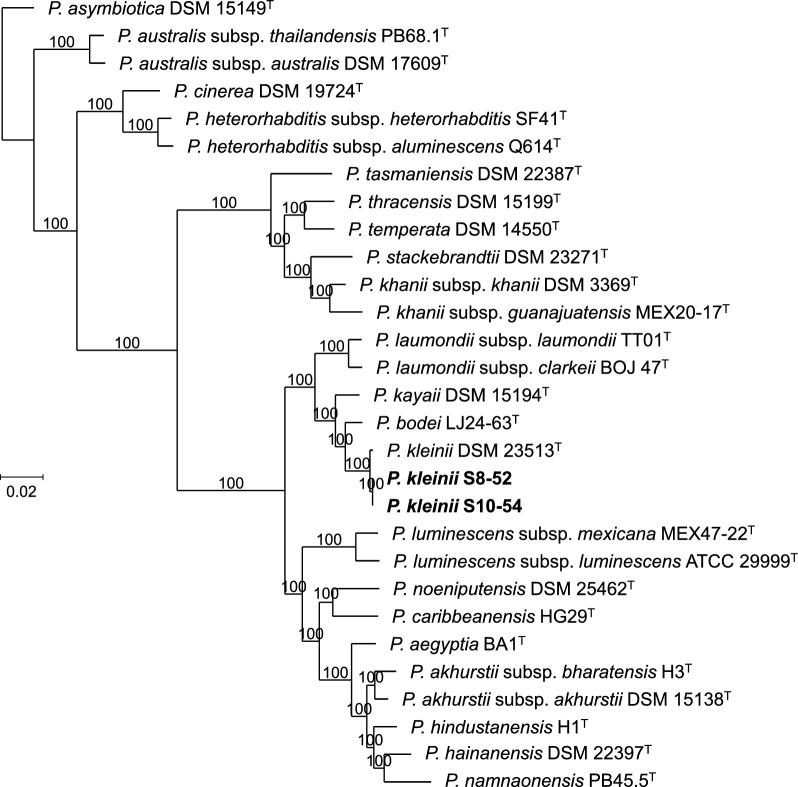
Phylogenetic reconstruction based on core genome sequences of the different *Photorhabdus* species. Bar represents average nucleotide substitutions per sequence position. Numbers at the nodes represent SH-like branch supports. Accession numbers of the genome sequences used for the reconstruction are presented in Table S3

Based on phylogenomic reconstructions using whole-genome sequences and on the sequence similarity values, *H. americana* n. sp. S8 and *H. americana* n. sp. S10 nematodes maintain symbiotic relationships with *Photorhabdus kleinii* (Fig. 13). Digital DNA–DNA hybridization (dDDH) values between S8-52 and S10-54, which are the symbiotic bacterial strains isolated from *H. americana* n. sp. S8 and *H. americana* n. sp. S10, respectively, are 100%, between S8-52 and *P. kleinii* DSM 23513^T^ are 94%, and between S10-54 and *P. kleinii* DSM 23513^T^ are also 94% (Fig. S8). When dDDH values between two strains are higher than 79%, the two strains are considered conspecific [97, 98].

## Authors’ note on the recent proposal of *Heterorhabditis alii* as a novel species

Recently, Shamseldean et al. [44] proposed *Heterorhabditis alii* Shamseldean, Abo-Shady, El-Awady & Heikal, 2024 as a novel species. However, the morphology and the morphometry of the studied specimens closely align with the type population of *H. indica* Poinar, Karunakar & David, 1992. SEM photographs of juveniles show transverse ridges on the ventral side, which are likely artifacts of the specimen preparation process. Unfortunately, no photographs of males were provided. On the other hand, the ITS sequence provided (NCBI accession OP555450) is of low quality and appears to be chimeric. Nevertheless, in the phylogenetic tree, *H. alii* clusters with *H. indica* and *H. hawaiiensis* Gardner, Stock & Kaya, 1994 (currently considered a junior synonym of *H. indica*). Based on these findings, *H. alii* is declared *species inquirenda*. Additional morphological and molecular support should be provided by the original descriptors to further support its novel species status.

## Conclusions

Given the morphological and morphometric differences, the distinct phylogenetic placement, and the reproductive isolation, the nematode isolates S8 and S10 represent a novel species, which we named *H. americana* n. sp.

## Supplementary Information

Additional file 1.

## Data Availability

The gene sequences obtained in this study were deposited in the GenBank database under the accession numbers given in Additional file 1: Tables S2 and S3. Data supporting the conclusions of this article are included within the article. The datasets used and/or analyzed during the current study are available from the corresponding author upon reasonable request.

## Abbreviations

GP: Genital papilla
IJs: Infective juveniles
ITS: Internal transcribed spacer
LB: Luria–Bertani
LM: Light microscopy
NCBI: National Center for Biotechnology Information
SEM: Scanning electron microscopy
