# Supplementary material for: Description of Heterorhabditis americana n. sp. (Rhabditida, Heterorhabditidae), a new entomopathogenic nematode species isolated in North America
Source: Parasit Vectors. 2025 Mar 11;18:101. doi: 10.1186/s13071-025-06702-5 (PMC11899345; doi:10.1186/s13071-025-06702-5)
Supplement: Supplementary file 1 — Additional file 1. [file 13071_2025_6702_MOESM1_ESM.pdf]

-SUPPLEMENTARY MATERIAL-

Description of *Heterorhabditis americana* n. sp. (Rhabditida, Heterorhabditidae), a new entomopathogenic nematode species isolated in North America

Ricardo A. R. Machado[1*], Joaquín Abolafia[2+], María-Cristina Robles[2+], Alba N. Ruiz-Cuenca[2+], Ebrahim Shokoohi[3+], Aashaq Hussain Bhat[1,4+], Vladimír Půža[5,6], Xi Zhang[7], Matthias Erb[7], Christelle A. M. Robert[7], Bruce Hibbard[8]

[1]*Experimental Biology Research Group. Institute of Biology. Faculty of Sciences. University of Neuchâtel. Rue Emile–Argand 11, 2000 Neuchâtel, Switzerland.*

[2]*Departamento de Biología Animal, Biología Vegetal y Ecología, Universidad de Jaén, Campus 'Las Lagunillas' s/n, Edificio B3, 23071 Jaén, Spain.*

[3]*Department of Biochemistry, Microbiology and Biotechnology, University of Limpopo, Private Bag X1106, Sovenga 0727, South Africa.*

[4]*University Centre for Research and Development and Department of Bioscience, Chandigarh University, Mohali 140413, Punjab, India*

[5]*Institute of Entomology, Biology centre of the Czech Academy of Sciences, CAS, 37005 České Budějovice, Czech Republic.*

[6]*Faculty of Agriculture and Technology, University of South Bohemia, 37005 České Budějovice, Czech Republic.*

[7]*Institute of Plant Sciences, University of Bern, Altenbergrain 21, 3013 Bern, Switzerland*

[8]*Plant Genetics Research Unit, United States Department of Agriculture (USDA)-Agricultural Research Service, Columbia, MO, United States.*

[+]: These authors contributed equally to this study.

**Correspondence:* Ricardo A. R. Machado. Emile–Argand 11, 2000 Neuchâtel, Switzerland. Email address: rarm.machado@gmail.com

SUPPLEMENTARY FIGURES

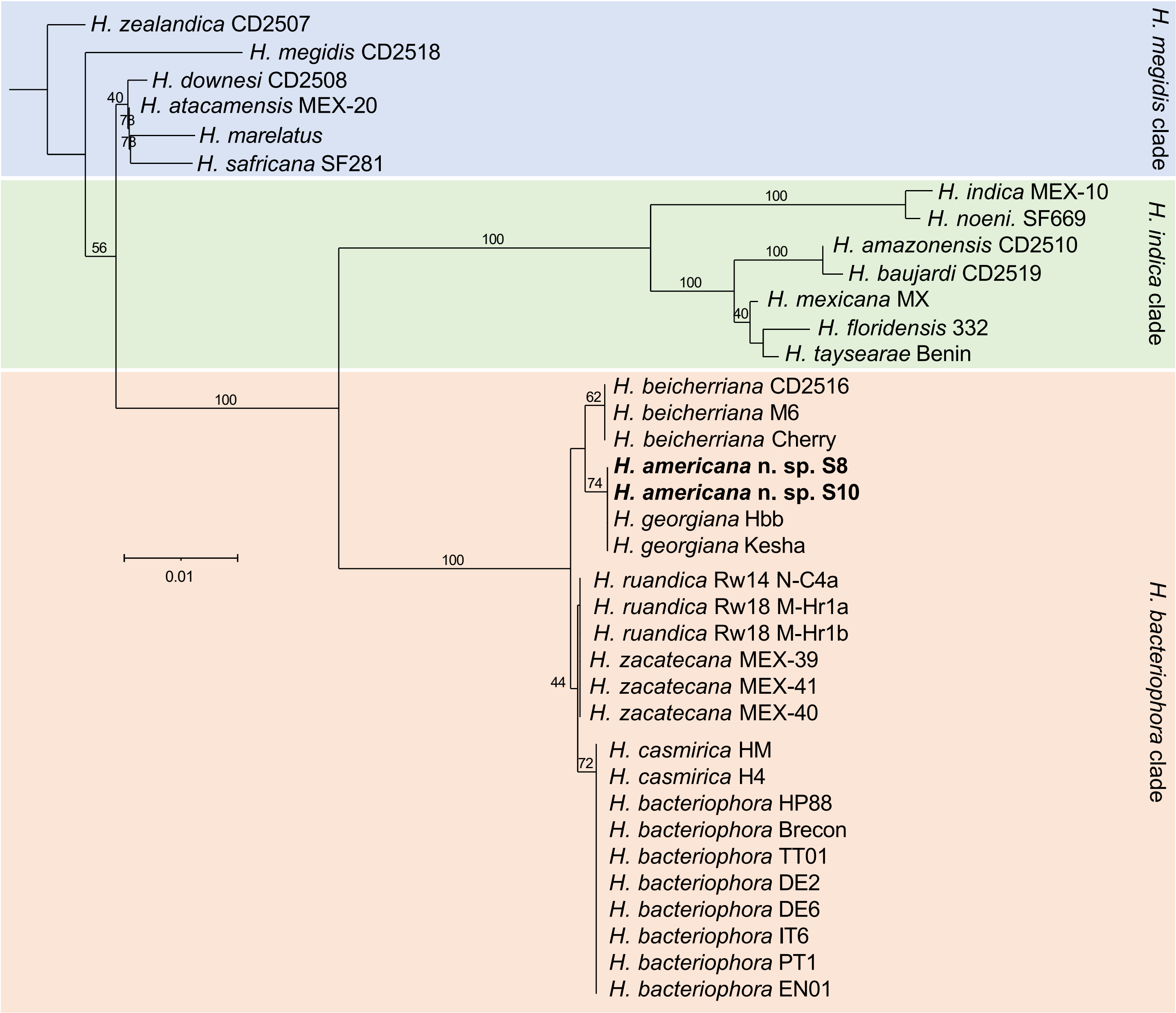

Figure S1. Maximum-likelihood phylogenetic tree reconstructed from the nucleotide sequences of the D2-D3 expansion segments of the 28S rRNA gene. A total of 554 nucleotide positions, flanked by primers D2A and D2B, were analyzed. Accession numbers of the nucleotide sequences used for the analyses are shown in Table S2. Numbers at nodes represent bootstrap values based on 100 replications. Bars represent average nucleotide substitutions per sequence position.

	H. zealandica CD2507	*H. downesi* CD2508	*H. megidis* CD2518	*H. marelatus*	*H. safricana* SF281	*H. atacamensis* MEX-20	*H. noenieputensis* SF669	*H. indica* LN2	*H. baujardi* CD2519	*H. amazonensis* CD2510	*H. mexicana*	*H. floridensis* 332	*H. taysearae* Benin	***H. americana* n. sp. S8**	***H. americana* n. sp. S10**	*H. georgiana* Kesha	*H. georgiana* Hbb	*H. beicherriana* M6	*H. beicherriana* Cherry	*H. beicherriana* CD2516	*H. ruandica* Rw14_N-C4a	*H. ruandica* Rw18_M-Hr1b	*H. ruandica* Rw18_M-Hr1a	*H. zacatecana* MEX-41	*H. zacatecana* MEX-40	*H. zacatecana* MEX-39	*H. casmirica* HM	*H. casmirica* H4	*H. bacteriophora* Brecon	*H. bacteriophora* HP88	*H. bacteriophora* DE2	*H. bacteriophora* DE6	*H. bacteriophora* EN01	*H. bacteriophora* IT6	*H. bacteriophora* TT01	*H. bacteriophora* PT1
H. zealandica CD2507	ID	90.0	93.0	91.2	91.5	90.0	86.5	86.5	89.7	88.0	88.6	88.3	88.0	91.5	91.5	91.2	91.5	92.4	92.4	92.4	90.9	90.6	90.6	90.3	90.9	90.9	91.2	91.2	90.6	90.9	90.9	90.9	90.9	90.9	90.9	90.9
H. downesi CD2508	90.0	ID	93.8	90.0	88.9	89.2	84.5	86.2	89.7	87.7	88.0	88.0	88.0	91.2	91.2	92.1	92.4	90.9	91.2	91.2	91.2	90.9	90.9	90.9	90.6	90.6	87.7	87.7	89.5	89.7	89.7	89.7	89.7	89.7	89.7	89.7
H. megidis CD2518	93.0	93.8	ID	91.8	90.9	90.3	84.8	88.0	89.7	87.7	88.6	88.0	88.0	92.1	92.1	93.0	93.2	92.1	92.1	92.1	91.8	91.5	91.5	90.6	90.9	90.9	89.7	89.7	90.3	90.6	90.6	90.6	90.6	90.6	90.6	90.6
H. marelatus	91.2	90.0	91.8	ID	91.5	92.4	85.4	86.0	87.7	86.2	86.8	86.0	86.2	90.6	90.6	90.6	90.6	91.2	90.9	90.9	89.7	89.5	89.5	89.2	89.2	89.2	87.7	87.7	88.3	88.0	88.3	88.3	88.3	88.3	88.3	88.3
H. safricana SF281	91.5	88.9	90.9	91.5	ID	91.5	85.7	85.1	88.6	88.0	87.4	87.4	87.7	89.7	89.7	90.0	89.7	90.3	90.0	90.0	89.7	89.5	89.5	88.9	89.5	89.5	88.8	88.8	88.3	87.7	88.0	88.0	88.0	88.0	88.0	88.0
H. atacamensis MEX-20	90.0	89.2	90.3	92.4	91.5	ID	86.5	86.2	88.9	87.4	86.2	85.7	86.5	89.7	89.7	90.3	90.3	89.5	89.2	89.2	88.6	88.3	88.3	88.0	88.0	88.0	87.7	87.7	87.7	87.4	87.7	87.7	87.7	87.7	87.7	87.7
H. noenieputensis SF669	86.5	84.5	84.8	85.4	85.7	86.5	ID	89.2	86.8	86.0	86.8	86.2	87.4	84.8	84.8	85.4	85.4	86.0	85.7	85.7	85.4	85.1	85.1	85.1	85.1	85.1	84.8	84.8	87.1	86.8	87.1	87.1	87.1	87.1	87.1	87.1
H. indica LN2	86.5	86.2	88.0	86.0	85.1	86.2	89.2	ID	88.3	85.7	86.8	87.4	85.7	86.2	86.2	86.2	86.2	85.7	85.4	85.4	84.2	83.9	83.9	83.6	84.2	84.2	83.3	83.3	85.4	85.4	85.4	85.4	85.4	85.4	85.4	85.4
H. baujardi CD2519	89.7	89.7	89.7	87.7	88.6	88.9	86.8	88.3	ID	92.1	94.4	92.7	94.4	88.0	88.0	89.2	89.2	88.0	88.3	88.3	87.1	86.8	86.8	86.5	86.5	86.5	86.2	86.2	88.6	88.6	88.6	88.6	88.6	88.6	88.6	88.6
H. amazonensis CD2510	88.0	87.7	87.7	86.2	88.0	87.4	86.0	85.7	92.1	ID	92.4	90.6	91.2	87.1	87.1	88.6	88.9	86.2	86.5	86.5	86.0	85.7	85.7	86.0	86.0	86.0	86.5	86.5	87.7	87.7	88.0	88.0	88.0	88.0	88.0	88.0
H. mexicana	88.6	88.0	88.6	86.8	87.4	86.2	86.8	86.8	94.4	92.4	ID	97.0	96.5	87.4	87.4	88.3	88.3	87.1	87.4	87.4	87.1	86.8	86.8	87.1	87.1	87.1	87.1	87.1	89.5	89.5	89.5	89.5	89.5	89.5	89.5	89.5
H. floridensis 332	88.3	88.0	88.0	86.0	87.4	85.7	86.2	87.4	92.7	90.6	97.0	ID	95.3	86.5	86.5	87.4	87.4	87.1	87.4	87.4	86.2	86.0	86.0	86.2	86.2	86.2	86.5	86.5	88.0	88.0	88.0	88.0	88.0	88.0	88.0	88.0
H. taysearae Benin	88.0	88.0	88.0	86.2	87.7	86.5	87.4	85.7	94.4	91.2	96.5	95.3	ID	86.8	86.8	88.0	88.0	88.0	88.3	88.3	87.4	87.1	87.1	87.4	86.8	86.8	87.1	87.1	89.5	89.2	89.5	89.5	89.5	89.5	89.5	89.5
***H. americana* n. sp. S8**	91.5	91.2	92.1	90.6	89.7	89.7	84.8	86.2	88.0	87.1	87.4	86.5	86.8	ID	100	96.7	97.0	93.5	93.2	93.2	92.1	91.8	91.8	92.1	92.4	92.4	91.5	91.5	92.4	92.4	92.7	92.7	92.7	92.7	92.7	92.7
***H. americana* n. sp. S10**	91.5	91.2	92.1	90.6	89.7	89.7	84.8	86.2	88.0	87.1	87.4	86.5	86.8	100	ID	96.7	97.0	93.5	93.2	93.2	92.1	91.8	91.8	92.1	92.4	92.4	91.5	91.5	92.4	92.4	92.7	92.7	92.7	92.7	92.7	92.7
H. georgiana Kesha	91.2	92.1	93.0	90.6	90.0	90.3	85.4	86.2	89.2	88.6	88.3	87.4	88.0	96.7	96.7	ID	99.7	93.2	93.5	93.5	93.0	92.7	92.7	92.4	92.7	92.7	92.3	92.3	93.2	92.7	93.0	93.0	93.0	93.0	93.0	93.0
H. georgiana Hbb	91.5	92.4	93.2	90.6	89.7	90.3	85.4	86.2	89.2	88.9	88.3	87.4	88.0	97.0	97.0	99.7	ID	93.5	93.8	93.8	93.2	93.0	93.0	92.7	93.0	93.0	92.1	92.1	93.0	93.0	93.2	93.2	93.2	93.2	93.2	93.2
H. beicherriana M6	92.4	90.9	92.1	91.2	90.3	89.5	86.0	85.7	88.0	86.2	87.1	87.1	88.0	93.5	93.5	93.2	93.5	ID	99.4	99.4	95.6	95.3	95.3	94.1	93.8	93.8	93.2	93.2	93.5	93.5	93.8	93.8	93.8	93.8	93.8	93.8
H. beicherriana Cherry	92.4	91.2	92.1	90.9	90.0	89.2	85.7	85.4	88.3	86.5	87.4	87.4	88.3	93.2	93.2	93.5	93.8	99.4	ID	100	95.0	94.7	94.7	93.8	93.5	93.5	93.2	93.2	93.2	93.2	93.5	93.5	93.5	93.5	93.5	93.5
H. beicherriana CD2516	92.4	91.2	92.1	90.9	90.0	89.2	85.7	85.4	88.3	86.5	87.4	87.4	88.3	93.2	93.2	93.5	93.8	99.4	100	ID	95.0	94.7	94.7	93.8	93.5	93.5	93.2	93.2	93.2	93.2	93.5	93.5	93.5	93.5	93.5	93.5
H. ruandica Rw14_N-C4a	90.9	91.2	91.8	89.7	89.7	88.6	85.4	84.2	87.1	86.0	87.1	86.2	87.4	92.1	92.1	93.0	93.2	95.6	95.0	95.0	ID	99.7	99.7	98.2	97.9	97.9	94.4	94.4	94.4	94.4	94.7	94.7	94.7	94.7	94.7	94.7
H. ruandica Rw18_M-Hr1b	90.6	90.9	91.5	89.5	89.5	88.3	85.1	83.9	86.8	85.7	86.8	86.0	87.1	91.8	91.8	92.7	93.0	95.3	94.7	94.7	99.7	ID	100	97.9	97.6	97.6	94.7	94.7	94.1	94.1	94.4	94.4	94.4	94.4	94.4	94.4
H. ruandica Rw18_M-Hr1a	90.6	90.9	91.5	89.5	89.5	88.3	85.1	83.9	86.8	85.7	86.8	86.0	87.1	91.8	91.8	92.7	93.0	95.3	94.7	94.7	99.7	100	ID	97.9	97.6	97.6	94.7	94.7	94.1	94.1	94.4	94.4	94.4	94.4	94.4	94.4
H. zacatecana MEX-41	90.3	90.9	90.6	89.2	88.9	88.0	85.1	83.6	86.5	86.0	87.1	86.2	87.4	92.1	92.1	92.4	92.7	94.1	93.8	93.8	98.2	97.9	97.9	ID	99.1	99.1	93.5	93.5	93.8	93.8	94.1	94.1	94.1	94.1	94.1	94.1
H. zacatecana MEX-40	90.9	90.6	90.9	89.2	89.5	88.0	85.1	84.2	86.5	86.0	87.1	86.2	86.8	92.4	92.4	92.7	93.0	93.8	93.5	93.5	97.9	97.6	97.6	99.1	ID	100	93.8	93.8	94.1	94.1	94.4	94.4	94.4	94.4	94.4	94.4
H. zacatecana MEX-39	90.9	90.6	90.9	89.2	89.5	88.0	85.1	84.2	86.5	86.0	87.1	86.2	86.8	92.4	92.4	92.7	93.0	93.8	93.5	93.5	97.9	97.6	97.6	99.1	100	ID	93.8	93.8	94.1	94.1	94.4	94.4	94.4	94.4	94.4	94.4
H. casmirica HM	91.2	87.7	89.7	87.7	88.8	87.7	84.8	83.3	86.2	86.5	87.1	86.5	87.1	91.5	91.5	92.3	92.1	93.2	93.2	93.2	94.4	94.7	94.7	93.5	93.8	93.8	ID	100	95.3	94.7	95.0	95.0	95.0	95.0	95.0	95.0
H. casmirica H4	91.2	87.7	89.7	87.7	88.8	87.7	84.8	83.3	86.2	86.5	87.1	86.5	87.1	91.5	91.5	92.3	92.1	93.2	93.2	93.2	94.4	94.7	94.7	93.5	93.8	93.8	100	ID	95.3	94.7	95.0	95.0	95.0	95.0	95.0	95.0
H. bacteriophora Brecon	90.6	89.5	90.3	88.3	88.3	87.7	87.1	85.4	88.6	87.7	89.5	88.0	89.5	92.4	92.4	93.2	93.0	93.5	93.2	93.2	94.4	94.1	94.1	93.8	94.1	94.1	95.3	95.3	ID	99.4	99.7	99.7	99.7	99.7	99.7	99.7
H. bacteriophora HP88	90.9	89.7	90.6	88.0	87.7	87.4	86.8	85.4	88.6	87.7	89.5	88.0	89.2	92.4	92.4	92.7	93.0	93.5	93.2	93.2	94.4	94.1	94.1	93.8	94.1	94.1	94.7	94.7	99.4	ID	99.7	99.7	99.7	99.7	99.7	99.7
H. bacteriophora DE2	90.9	89.7	90.6	88.3	88.0	87.7	87.1	85.4	88.6	88.0	89.5	88.0	89.5	92.7	92.7	93.0	93.2	93.8	93.5	93.5	94.7	94.4	94.4	94.1	94.4	94.4	95.0	95.0	99.7	99.7	ID	100	100	100	100	100
H. bacteriophora DE6	90.9	89.7	90.6	88.3	88.0	87.7	87.1	85.4	88.6	88.0	89.5	88.0	89.5	92.7	92.7	93.0	93.2	93.8	93.5	93.5	94.7	94.4	94.4	94.1	94.4	94.4	95.0	95.0	99.7	99.7	100	ID	100	100	100	100
H. bacteriophora EN01	90.9	89.7	90.6	88.3	88.0	87.7	87.1	85.4	88.6	88.0	89.5	88.0	89.5	92.7	92.7	93.0	93.2	93.8	93.5	93.5	94.7	94.4	94.4	94.1	94.4	94.4	95.0	95.0	99.7	99.7	100	100	ID	100	100	100
H. bacteriophora IT6	90.9	89.7	90.6	88.3	88.0	87.7	87.1	85.4	88.6	88.0	89.5	88.0	89.5	92.7	92.7	93.0	93.2	93.8	93.5	93.5	94.7	94.4	94.4	94.1	94.4	94.4	95.0	95.0	99.7	99.7	100	100	100	ID	100	100
H. bacteriophora TT01	90.9	89.7	90.6	88.3	88.0	87.7	87.1	85.4	88.6	88.0	89.5	88.0	89.5	92.7	92.7	93.0	93.2	93.8	93.5	93.5	94.7	94.4	94.4	94.1	94.4	94.4	95.0	95.0	99.7	99.7	100	100	100	100	ID	100
H. bacteriophora PT1	90.9	89.7	90.6	88.3	88.0	87.7	87.1	85.4	88.6	88.0	89.5	88.0	89.5	92.7	92.7	93.0	93.2	93.8	93.5	93.5	94.7	94.4	94.4	94.1	94.4	94.4	95.0	95.0	99.7	99.7	100	100	100	100	100	ID

Figure S2. Pairwise comparisons of nucleotide similarity (%) values for the sequences of the cytochrome c oxidase I (*cox-1*) gene of different *Heterorhabditis* species. A total of 344 nucleotide positions, flanked by primers HCF and HCR, were analyzed. Accession numbers of gene sequences used are shown in Table S2.

	H. bacteriophora PT1	*H. bacteriophora* TT01	*H. bacteriophora* IT6	*H. bacteriophora* EN01	*H. bacteriophora* DE6	*H. bacteriophora* DE2	*H. bacteriophora* HP88	*H. bacteriophora* Brecon	*H. casmirica* H4	*H. casmirica* HM	*H. zacatecana* MEX-39	*H. zacatecana* MEX-40	*H. zacatecana* MEX-41	*H. ruandica* Rw18_M-Hr1a	*H. ruandica* Rw18_M-Hr1b	*H. ruandica* Rw14_N-C4a	*H. beicherriana* CD2516	*H. beicherriana* Cherry	*H. beicherriana* M6	*H. georgiana* Hbb	*H. georgiana* Kesha	***H. americana* n. sp. S10**	***H. americana* n. sp. S8**	*H. taysearae* Benin	*H. floridensis* 332	*H. mexicana*	*H. amazonensis* CD2510	*H. baujardi* CD2519	*H. indica* LN2	*H. noenieputensis* SF669	*H. atacamensis* MEX-20	*H. safricana* SF281	*H. marelatus*	*H. megidis* CD2518	*H. downesi* CD2508	*H. zealandica* CD2507
H. zealandica CD2507	31	31	31	31	31	31	31	32	30	30	31	31	33	32	32	31	26	26	26	29	30	29	29	41	40	39	41	35	46	46	34	29	30	24	34	ID
H. downesi CD2508	35	35	35	35	35	35	35	36	42	42	32	32	31	31	31	30	30	30	31	26	27	30	30	41	41	41	42	35	47	53	37	38	34	21	ID	34
H. megidis CD2518	32	32	32	32	32	32	32	33	35	35	31	31	32	29	29	28	27	27	27	23	24	27	27	41	41	39	42	35	41	52	33	31	28	ID	21	24
H. marelatus	40	40	40	40	40	40	41	40	42	42	37	37	37	36	36	35	31	31	30	32	32	32	32	47	48	45	47	42	48	50	26	29	ID	28	34	30
H. safricana SF281	41	41	41	41	41	41	42	40	38	38	36	36	38	36	36	35	34	34	33	35	34	35	35	42	43	43	41	39	51	49	29	ID	29	31	38	29
H. atacamensis MEX-20	42	42	42	42	42	42	43	42	42	42	41	41	41	40	40	39	37	37	36	33	33	35	35	46	49	47	43	38	47	46	ID	29	26	33	37	34
H. noenieputensis SF669	44	44	44	44	44	44	45	44	52	52	51	51	51	51	51	50	49	49	48	50	50	52	52	43	47	45	48	45	37	ID	46	49	50	52	53	46
H. indica LN2	50	50	50	50	50	50	50	50	57	57	54	54	56	55	55	54	50	50	49	47	47	47	47	49	43	45	49	40	ID	37	47	51	48	41	47	46
H. baujardi CD2519	39	39	39	39	39	39	39	39	47	47	46	46	46	45	45	44	40	40	41	37	37	41	41	19	25	19	27	ID	40	45	38	39	42	35	35	35
H. amazonensis CD2510	41	41	41	41	41	41	42	42	46	46	48	48	48	49	49	48	46	46	47	38	39	44	44	30	32	26	ID	27	49	48	43	41	47	42	42	41
H. mexicana	36	36	36	36	36	36	36	36	44	44	44	44	44	45	45	44	43	43	44	40	40	43	43	12	10	ID	26	19	45	45	47	43	45	39	41	39
H. floridensis 332	41	41	41	41	41	41	41	41	46	46	47	47	47	48	48	47	43	43	44	43	43	46	46	16	ID	10	32	25	43	47	49	43	48	41	41	40
H. taysearae Benin	36	36	36	36	36	36	37	36	44	44	45	45	43	44	44	43	40	40	41	41	41	45	45	ID	16	12	30	19	49	43	46	42	47	41	41	41
***H. americana* n. sp. S8**	25	25	25	25	25	25	26	26	29	29	26	26	27	28	28	27	23	23	22	10	11	0	ID	45	46	43	44	41	47	52	35	35	32	27	30	29
***H. americana* n. sp. S10**	25	25	25	25	25	25	26	26	29	29	26	26	27	28	28	27	23	23	22	10	11	ID	0	45	46	43	44	41	47	52	35	35	32	27	30	29
H. georgiana Kesha	24	24	24	24	24	24	25	23	26	26	25	25	26	25	25	24	22	22	23	1	ID	11	11	41	43	40	39	37	47	50	33	34	32	24	27	30
H. georgiana Hbb	23	23	23	23	23	23	24	24	27	27	24	24	25	24	24	23	21	21	22	ID	1	10	10	41	43	40	38	37	47	50	33	35	32	23	26	29
H. beicherriana M6	21	21	21	21	21	21	22	22	23	23	21	21	20	16	16	15	2	2	ID	22	23	22	22	41	44	44	47	41	49	48	36	33	30	27	31	26
H. beicherriana Cherry	22	22	22	22	22	22	23	23	23	23	22	22	21	18	18	17	0	ID	2	21	22	23	23	40	43	43	46	40	50	49	37	34	31	27	30	26
H. beicherriana CD2516	22	22	22	22	22	22	23	23	23	23	22	22	21	18	18	17	ID	0	2	21	22	23	23	40	43	43	46	40	50	49	37	34	31	27	30	26
H. ruandica Rw14_N-C4a	18	18	18	18	18	18	19	19	19	19	7	7	6	1	1	ID	17	17	15	23	24	27	27	43	47	44	48	44	54	50	39	35	35	28	30	31
H. ruandica Rw18_M-Hr1b	19	19	19	19	19	19	20	20	18	18	8	8	7	0	ID	1	18	18	16	24	25	28	28	44	48	45	49	45	55	51	40	36	36	29	31	32
H. ruandica Rw18_M-Hr1a	19	19	19	19	19	19	20	20	18	18	8	8	7	ID	0	1	18	18	16	24	25	28	28	44	48	45	49	45	55	51	40	36	36	29	31	32
H. zacatecana MEX-41	20	20	20	20	20	20	21	21	22	22	3	3	ID	7	7	6	21	21	20	25	26	27	27	43	47	44	48	46	56	51	41	38	37	32	31	33
H. zacatecana MEX-40	19	19	19	19	19	19	20	20	21	21	0	ID	3	8	8	7	22	22	21	24	25	26	26	45	47	44	48	46	54	51	41	36	37	31	32	31
H. zacatecana MEX-39	19	19	19	19	19	19	20	20	21	21	ID	0	3	8	8	7	22	22	21	24	25	26	26	45	47	44	48	46	54	51	41	36	37	31	32	31
H. casmirica HM	17	17	17	17	17	17	18	16	0	ID	21	21	22	18	18	19	23	23	23	27	26	29	29	44	46	44	46	47	57	52	42	38	42	35	42	30
H. casmirica H4	17	17	17	17	17	17	18	16	ID	0	21	21	22	18	18	19	23	23	23	27	26	29	29	44	46	44	46	47	57	52	42	38	42	35	42	30
H. bacteriophora Brecon	1	1	1	1	1	1	2	ID	16	16	20	20	21	20	20	19	23	23	22	24	23	26	26	36	41	36	42	39	50	44	42	40	40	33	36	32
H. bacteriophora HP88	1	1	1	1	1	1	ID	2	18	18	20	20	21	20	20	19	23	23	22	24	25	26	26	37	41	36	42	39	50	45	43	42	41	32	35	31
H. bacteriophora DE2	0	0	0	0	0	ID	1	1	17	17	19	19	20	19	19	18	22	22	21	23	24	25	25	36	41	36	41	39	50	44	42	41	40	32	35	31
H. bacteriophora DE6	0	0	0	0	ID	0	1	1	17	17	19	19	20	19	19	18	22	22	21	23	24	25	25	36	41	36	41	39	50	44	42	41	40	32	35	31
H. bacteriophora EN01	0	0	0	ID	0	0	1	1	17	17	19	19	20	19	19	18	22	22	21	23	24	25	25	36	41	36	41	39	50	44	42	41	40	32	35	31
H. bacteriophora IT6	0	0	ID	0	0	0	1	1	17	17	19	19	20	19	19	18	22	22	21	23	24	25	25	36	41	36	41	39	50	44	42	41	40	32	35	31
H. bacteriophora TT01	0	ID	0	0	0	0	1	1	17	17	19	19	20	19	19	18	22	22	21	23	24	25	25	36	41	36	41	39	50	44	42	41	40	32	35	31
H. bacteriophora PT1	ID	0	0	0	0	0	1	1	17	17	19	19	20	19	19	18	22	22	21	23	24	25	25	36	41	36	41	39	50	44	42	41	40	32	35	31

Figure S3. Pairwise comparisons of the number of nucleotide differences in the sequences of the cytochrome c oxidase I (*cox-1*) gene of different *Heterorhabditis* species. A total of 344 nucleotide positions, flanked by primers HCF and HCR, were analyzed. Accession numbers of gene sequences used are shown in Table S2.

	H. zealandica CD2507	*H. megidis* CD2518	*H. downesi* CD2508	*H. marelatus* OH10	*H. safricana* SF281	*H. atacamensis* MEX-20	*H. noenieputensis* SF669	*H. indica* CD2525	*H. amazonensis* CD2510	*H. baujardi* CD2519	*H. mexicana* Mexican	*H. floridensis* 332	*H. tayserae* Benin	*H. beicherriana* CD2516	*H. beicherriana* M6	*H. beicherriana* Cherry	*H. georgiana* Kesha	*H. georgiana* Hbb	***H. americana* n. sp. S10**	***H. americana* n. sp. S8**	*H. zacatecana* MEX-40	*H. zacatecana* MEX-39	*H. zacatecana* MEX-41	*H. ruandica* Rw14_N-C4a	*H. ruandica* Rw18_M-Hr1b	*H. ruandica* Rw18_M-Hr1a	*H. casmirica* H4	*H. casmirica* HM	*H. bacteriophora* TT01	*H. bacteriophora* HP88	*H. bacteriophora* Brecon	*H. bacteriophora* DE2	*H. bacteriophora* DE6	*H. bacteriophora* IT6	*H. bacteriophora* EN01	*H. bacteriophora* PT1
H. zealandica CD2507	ID	84.1	88.2	88.5	87.8	88.5	67.6	67.7	69.1	68.8	68.0	68.0	68.2	74.2	74.2	74.2	74.1	74.1	74.0	74.0	74.0	74.0	74.0	73.8	73.8	73.8	74.3	74.3	74.4	74.4	74.4	74.4	74.4	74.4	74.4	74.4
H. megidis CD2518	84.1	ID	92.7	89.4	89.8	90.2	66.4	66.5	67.9	67.8	67.0	66.8	67.5	74.3	74.3	74.3	74.2	74.2	74.3	74.3	74.6	74.6	74.6	74.4	74.4	74.4	74.5	74.5	74.6	74.6	74.6	74.6	74.6	74.6	74.6	74.6
H. downesi CD2508	88.2	92.7	ID	94.2	94.7	95.3	69.5	69.6	70.8	70.8	69.7	69.7	70.2	77.7	77.7	77.7	77.4	77.4	77.5	77.5	77.4	77.4	77.4	77.1	77.1	77.1	77.5	77.5	77.7	77.7	77.7	77.7	77.7	77.7	77.7	77.7
H. marelatus OH10	88.5	89.4	94.2	ID	96.6	97.0	70.1	70.1	72.2	71.9	70.7	70.9	71.0	78.5	78.5	78.5	78.2	78.2	78.3	78.3	78.5	78.5	78.5	78.2	78.2	78.2	78.5	78.5	78.6	78.6	78.6	78.6	78.6	78.6	78.6	78.6
H. safricana SF281	87.8	89.8	94.7	96.6	ID	98.1	70.5	70.5	71.6	71.4	70.2	70.3	70.7	78.2	78.2	78.2	77.9	77.9	78.0	78.0	77.9	77.9	77.9	77.7	77.7	77.7	78.1	78.1	78.2	78.2	78.2	78.2	78.2	78.2	78.2	78.2
H. atacamensis MEX-20	88.5	90.2	95.3	97.0	98.1	ID	71.4	71.4	72.6	72.3	71.1	71.3	71.7	78.8	78.8	78.8	78.4	78.4	78.6	78.6	78.5	78.5	78.5	78.2	78.2	78.2	78.7	78.7	78.9	78.9	78.9	78.9	78.9	78.9	78.9	78.9
H. noenieputensis SF669	67.6	66.4	69.5	70.1	70.5	71.4	ID	98.0	84.7	84.9	84.2	84.3	84.1	71.0	71.0	71.0	71.0	71.0	70.9	70.9	70.7	70.7	70.7	70.8	70.8	70.8	70.8	70.8	70.8	70.8	70.8	70.8	70.8	70.8	70.8	70.8
H. indica CD2525	67.7	66.5	69.6	70.1	70.5	71.4	98.0	ID	85.0	85.1	84.5	84.3	84.3	70.8	70.8	70.8	70.9	70.9	70.8	70.8	70.6	70.6	70.6	70.6	70.6	70.6	70.7	70.7	70.7	70.7	70.7	70.7	70.7	70.7	70.7	70.7
H. amazonensis CD2510	69.1	67.9	70.8	72.2	71.6	72.6	84.7	85.0	ID	98.0	96.5	96.9	96.8	72.0	72.0	72.0	72.1	72.1	71.9	71.9	71.9	71.9	71.9	71.9	71.9	71.9	72.0	72.0	72.3	72.3	72.3	72.3	72.3	72.3	72.3	72.3
H. baujardi CD2519	68.8	67.8	70.8	71.9	71.4	72.3	84.9	85.1	98.0	ID	96.4	97.2	96.5	71.9	71.9	71.9	71.9	71.9	71.8	71.8	71.9	71.9	71.9	71.9	71.9	71.9	72.0	72.0	72.3	72.3	72.3	72.3	72.3	72.3	72.3	72.3
H. mexicana Mexican	68.0	67.0	69.7	70.7	70.2	71.1	84.2	84.5	96.5	96.4	ID	98.2	98.7	70.6	70.6	70.6	70.6	70.6	70.5	70.5	70.6	70.6	70.6	70.6	70.6	70.6	70.7	70.7	71.0	71.0	71.0	71.0	71.0	71.0	71.0	71.0
H. floridensis 332	68.0	66.8	69.7	70.9	70.3	71.3	84.3	84.3	96.9	97.2	98.2	ID	98.2	70.7	70.7	70.7	70.8	70.8	70.6	70.6	70.7	70.7	70.7	70.7	70.7	70.7	70.8	70.8	71.1	71.1	71.1	71.1	71.1	71.1	71.1	71.1
H. tayserae Benin	68.2	67.5	70.2	71.0	70.7	71.7	84.1	84.3	96.8	96.5	98.7	98.2	ID	70.6	70.6	70.6	70.6	70.6	70.5	70.5	70.2	70.2	70.2	70.2	70.2	70.2	70.3	70.3	70.6	70.6	70.6	70.6	70.6	70.6	70.6	70.6
H. beicherriana CD2516	74.2	74.3	77.7	78.5	78.2	78.8	71.0	70.8	72.0	71.9	70.6	70.7	70.6	ID	100	100	97.5	97.5	97.6	97.6	97.8	97.8	97.8	97.5	97.5	97.5	97.8	97.8	97.5	97.5	97.5	97.5	97.5	97.5	97.5	97.5
H. beicherriana M6	74.2	74.3	77.7	78.5	78.2	78.8	71.0	70.8	72.0	71.9	70.6	70.7	70.6	100	ID	100	97.5	97.5	97.6	97.6	97.8	97.8	97.8	97.5	97.5	97.5	97.8	97.8	97.5	97.5	97.5	97.5	97.5	97.5	97.5	97.5
H. beicherriana Cherry	74.2	74.3	77.7	78.5	78.2	78.8	71.0	70.8	72.0	71.9	70.6	70.7	70.6	100	100	ID	97.5	97.5	97.6	97.6	97.8	97.8	97.8	97.5	97.5	97.5	97.8	97.8	97.5	97.5	97.5	97.5	97.5	97.5	97.5	97.5
H. georgiana Kesha	74.1	74.2	77.4	78.2	77.9	78.4	71.0	70.9	72.1	71.9	70.6	70.8	70.6	97.5	97.5	97.5	ID	100	99.8	99.8	97.6	97.6	97.6	97.4	97.4	97.4	97.4	97.4	97.1	97.1	97.1	97.1	97.1	97.1	97.1	97.1
H. georgiana Hbb	74.1	74.2	77.4	78.2	77.9	78.4	71.0	70.9	72.1	71.9	70.6	70.8	70.6	97.5	97.5	97.5	100	ID	99.8	99.8	97.6	97.6	97.6	97.4	97.4	97.4	97.4	97.4	97.1	97.1	97.1	97.1	97.1	97.1	97.1	97.1
***H. americana* n. sp. S10**	74.0	74.3	77.5	78.3	78.0	78.6	70.9	70.8	71.9	71.8	70.5	70.6	70.5	97.6	97.6	97.6	99.8	99.8	ID	100	97.8	97.8	97.8	97.5	97.5	97.5	97.5	97.5	97.2	97.2	97.2	97.2	97.2	97.2	97.2	97.2
***H. americana* n. sp. S8**	74.0	74.3	77.5	78.3	78.0	78.6	70.9	70.8	71.9	71.8	70.5	70.6	70.5	97.6	97.6	97.6	99.8	99.8	100	ID	97.8	97.8	97.8	97.5	97.5	97.5	97.5	97.5	97.2	97.2	97.2	97.2	97.2	97.2	97.2	97.2
H. zacatecana MEX-40	74.0	74.6	77.4	78.5	77.9	78.5	70.7	70.6	71.9	71.9	70.6	70.7	70.2	97.8	97.8	97.8	97.6	97.6	97.8	97.8	ID	100	100	99.7	99.7	99.7	99.7	99.7	99.4	99.4	99.4	99.4	99.4	99.4	99.4	99.4
H. zacatecana MEX-39	74.0	74.6	77.4	78.5	77.9	78.5	70.7	70.6	71.9	71.9	70.6	70.7	70.2	97.8	97.8	97.8	97.6	97.6	97.8	97.8	100	ID	100	99.7	99.7	99.7	99.7	99.7	99.4	99.4	99.4	99.4	99.4	99.4	99.4	99.4
H. zacatecana MEX-41	74.0	74.6	77.4	78.5	77.9	78.5	70.7	70.6	71.9	71.9	70.6	70.7	70.2	97.8	97.8	97.8	97.6	97.6	97.8	97.8	100	100	ID	99.7	99.7	99.7	99.7	99.7	99.4	99.4	99.4	99.4	99.4	99.4	99.4	99.4
H. ruandica Rw14_N-C4a	73.8	74.4	77.1	78.2	77.7	78.2	70.8	70.6	71.9	71.9	70.6	70.7	70.2	97.5	97.5	97.5	97.4	97.4	97.5	97.5	99.7	99.7	99.7	ID	100	100	99.4	99.4	99.1	99.1	99.1	99.1	99.1	99.1	99.1	99.1
H. ruandica Rw18_M-Hr1b	73.8	74.4	77.1	78.2	77.7	78.2	70.8	70.6	71.9	71.9	70.6	70.7	70.2	97.5	97.5	97.5	97.4	97.4	97.5	97.5	99.7	99.7	99.7	100	ID	100	99.4	99.4	99.1	99.1	99.1	99.1	99.1	99.1	99.1	99.1
H. ruandica Rw18_M-Hr1a	73.8	74.4	77.1	78.2	77.7	78.2	70.8	70.6	71.9	71.9	70.6	70.7	70.2	97.5	97.5	97.5	97.4	97.4	97.5	97.5	99.7	99.7	99.7	100	100	ID	99.4	99.4	99.1	99.1	99.1	99.1	99.1	99.1	99.1	99.1
H. casmirica H4	74.3	74.5	77.5	78.5	78.1	78.7	70.8	70.7	72.0	72.0	70.7	70.8	70.3	97.8	97.8	97.8	97.4	97.4	97.5	97.5	99.7	99.7	99.7	99.4	99.4	99.4	ID	100	99.7	99.7	99.7	99.7	99.7	99.7	99.7	99.7
H. casmirica HM	74.3	74.5	77.5	78.5	78.1	78.7	70.8	70.7	72.0	72.0	70.7	70.8	70.3	97.8	97.8	97.8	97.4	97.4	97.5	97.5	99.7	99.7	99.7	99.4	99.4	99.4	100	ID	99.7	99.7	99.7	99.7	99.7	99.7	99.7	99.7
H. bacteriophora TT01	74.4	74.6	77.7	78.6	78.2	78.9	70.8	70.7	72.3	72.3	71.0	71.1	70.6	97.5	97.5	97.5	97.1	97.1	97.2	97.2	99.4	99.4	99.4	99.1	99.1	99.1	99.7	99.7	ID	100	100	100	100	100	100	100
H. bacteriophora HP88	74.4	74.6	77.7	78.6	78.2	78.9	70.8	70.7	72.3	72.3	71.0	71.1	70.6	97.5	97.5	97.5	97.1	97.1	97.2	97.2	99.4	99.4	99.4	99.1	99.1	99.1	99.7	99.7	100	ID	100	100	100	100	100	100
H. bacteriophora Brecon	74.4	74.6	77.7	78.6	78.2	78.9	70.8	70.7	72.3	72.3	71.0	71.1	70.6	97.5	97.5	97.5	97.1	97.1	97.2	97.2	99.4	99.4	99.4	99.1	99.1	99.1	99.7	99.7	100	100	ID	100	100	100	100	100
H. bacteriophora DE2	74.4	74.6	77.7	78.6	78.2	78.9	70.8	70.7	72.3	72.3	71.0	71.1	70.6	97.5	97.5	97.5	97.1	97.1	97.2	97.2	99.4	99.4	99.4	99.1	99.1	99.1	99.7	99.7	100	100	100	ID	100	100	100	100
H. bacteriophora DE6	74.4	74.6	77.7	78.6	78.2	78.9	70.8	70.7	72.3	72.3	71.0	71.1	70.6	97.5	97.5	97.5	97.1	97.1	97.2	97.2	99.4	99.4	99.4	99.1	99.1	99.1	99.7	99.7	100	100	100	100	ID	100	100	100
H. bacteriophora IT6	74.4	74.6	77.7	78.6	78.2	78.9	70.8	70.7	72.3	72.3	71.0	71.1	70.6	97.5	97.5	97.5	97.1	97.1	97.2	97.2	99.4	99.4	99.4	99.1	99.1	99.1	99.7	99.7	100	100	100	100	100	ID	100	100
H. bacteriophora EN01	74.4	74.6	77.7	78.6	78.2	78.9	70.8	70.7	72.3	72.3	71.0	71.1	70.6	97.5	97.5	97.5	97.1	97.1	97.2	97.2	99.4	99.4	99.4	99.1	99.1	99.1	99.7	99.7	100	100	100	100	100	100	ID	100
H. bacteriophora PT1	74.4	74.6	77.7	78.6	78.2	78.9	70.8	70.7	72.3	72.3	71.0	71.1	70.6	97.5	97.5	97.5	97.1	97.1	97.2	97.2	99.4	99.4	99.4	99.1	99.1	99.1	99.7	99.7	100	100	100	100	100	100	100	ID

Figure S4. Pairwise comparisons of the nucleotide similarity (%) values for the sequences of the internal transcribed spacer (ITS) region of the rRNA gene of different *Heterorhabditis* species. A total of 775 nucleotide positions, flanked by primers TW81 and AB28, were analyzed. Accession numbers of gene sequences used are shown in Table S2.

	H. bacteriophora PT1	*H. bacteriophora* EN01	*H. bacteriophora* IT6	*H. bacteriophora* DE6	*H. bacteriophora* DE2	*H. bacteriophora* Brecon	*H. bacteriophora* HP88	*H. bacteriophora* TT01	*H. casmirica* HM	*H. casmirica* H4	*H. ruandica* Rw18_M-Hr1a	*H. ruandica* Rw18_M-Hr1b	*H. ruandica* Rw14_N-C4a	*H. zacatecana* MEX-41	*H. zacatecana* MEX-39	*H. zacatecana* MEX-40	***H. americana* n. sp. S8**	***H. americana* n. sp. S10**	*H. georgiana* Hbb	*H. georgiana* Kesha	*H. beicherriana* Cherry	*H. beicherriana* M6	*H. beicherriana* CD2516	*H. tayserae* Benin	*H. floridensis* 332	*H. mexicana* Mexican	*H. baujardi* CD2519	*H. amazonensis* CD2510	*H. indica* CD2525	*H. noenieputensis* SF669	*H. atacamensis* MEX-20	*H. safricana* SF281	*H. marelatus* OH10	*H. downesi* CD2508	*H. megidis* CD2518	*H. zealandica* CD2507
H. zealandica CD2507	190	190	190	190	190	190	190	190	191	191	195	195	195	193	193	193	193	193	192	192	192	192	192	237	238	238	232	230	239	240	82	87	82	84	116	ID
H. megidis CD2518	191	191	191	191	191	191	191	191	192	192	193	193	193	191	191	191	193	193	194	194	194	194	194	246	251	250	244	243	252	253	71	74	77	52	ID	116
H. downesi CD2508	165	165	165	165	165	165	165	165	166	166	169	169	169	167	167	167	166	166	167	167	165	165	165	221	225	225	216	216	224	225	33	37	41	ID	52	84
H. marelatus OH10	158	158	158	158	158	158	158	158	159	159	161	161	161	159	159	159	160	160	161	161	159	159	159	215	216	217	208	206	221	221	21	24	ID	41	77	82
H. safricana SF281	161	161	161	161	161	161	161	161	162	162	165	165	165	163	163	163	162	162	163	163	161	161	161	217	220	221	212	210	218	218	13	ID	24	37	74	87
H. atacamensis MEX-20	156	156	156	156	156	156	156	156	157	157	161	161	161	159	159	159	158	158	159	159	157	157	157	210	213	214	205	203	211	211	ID	13	21	33	71	82
H. noenieputensis SF669	220	220	220	220	220	220	220	220	220	220	220	220	220	221	221	221	219	219	218	218	219	219	219	117	115	116	111	112	14	ID	211	218	221	225	253	240
H. indica CD2525	221	221	221	221	221	221	221	221	221	221	222	222	222	222	222	222	220	220	219	219	220	220	220	115	115	114	109	110	ID	14	211	218	221	224	252	239
H. amazonensis CD2510	209	209	209	209	209	209	209	209	211	211	212	212	212	212	212	212	211	211	210	210	211	211	211	23	22	25	14	ID	110	112	203	210	206	216	243	230
H. baujardi CD2519	209	209	209	209	209	209	209	209	211	211	212	212	212	212	212	212	212	212	211	211	212	212	212	25	20	26	ID	14	109	111	205	212	208	216	244	232
H. mexicana Mexican	219	219	219	219	219	219	219	219	221	221	222	222	222	222	222	222	222	222	221	221	222	222	222	9	13	ID	26	25	114	116	214	221	217	225	250	238
H. floridensis 332	218	218	218	218	218	218	218	218	220	220	221	221	221	221	221	221	221	221	220	220	221	221	221	13	ID	13	20	22	115	115	213	220	216	225	251	238
H. tayserae Benin	222	222	222	222	222	222	222	222	224	224	225	225	225	225	225	225	222	222	221	221	222	222	222	ID	13	9	25	23	115	117	210	217	215	221	246	237
H. beicherriana CD2516	18	18	18	18	18	18	18	18	16	16	18	18	18	16	16	16	17	17	18	18	0	0	ID	222	221	222	212	211	220	219	157	161	159	165	194	192
H. beicherriana M6	18	18	18	18	18	18	18	18	16	16	18	18	18	16	16	16	17	17	18	18	0	ID	0	222	221	222	212	211	220	219	157	161	159	165	194	192
H. beicherriana Cherry	18	18	18	18	18	18	18	18	16	16	18	18	18	16	16	16	17	17	18	18	ID	0	0	222	221	222	212	211	220	219	157	161	159	165	194	192
H. georgiana Kesha	21	21	21	21	21	21	21	21	19	19	19	19	19	17	17	17	1	1	0	ID	18	18	18	221	220	221	211	210	219	218	159	163	161	167	194	192
H. georgiana Hbb	21	21	21	21	21	21	21	21	19	19	19	19	19	17	17	17	1	1	ID	0	18	18	18	221	220	221	211	210	219	218	159	163	161	167	194	192
***H. americana* n. sp. S10**	20	20	20	20	20	20	20	20	18	18	18	18	18	16	16	16	0	ID	1	1	17	17	17	222	221	222	212	211	220	219	158	162	160	166	193	193
***H. americana* n. sp. S8**	20	20	20	20	20	20	20	20	18	18	18	18	18	16	16	16	ID	0	1	1	17	17	17	222	221	222	212	211	220	219	158	162	160	166	193	193
H. zacatecana MEX-40	4	4	4	4	4	4	4	4	2	2	2	2	2	0	0	ID	16	16	17	17	16	16	16	225	221	222	212	212	222	221	159	163	159	167	191	193
H. zacatecana MEX-39	4	4	4	4	4	4	4	4	2	2	2	2	2	0	ID	0	16	16	17	17	16	16	16	225	221	222	212	212	222	221	159	163	159	167	191	193
H. zacatecana MEX-41	4	4	4	4	4	4	4	4	2	2	2	2	2	ID	0	0	16	16	17	17	16	16	16	225	221	222	212	212	222	221	159	163	159	167	191	193
H. ruandica Rw14_N-C4a	6	6	6	6	6	6	6	6	4	4	0	0	ID	2	2	2	18	18	19	19	18	18	18	225	221	222	212	212	222	220	161	165	161	169	193	195
H. ruandica Rw18_M-Hr1b	6	6	6	6	6	6	6	6	4	4	0	ID	0	2	2	2	18	18	19	19	18	18	18	225	221	222	212	212	222	220	161	165	161	169	193	195
H. ruandica Rw18_M-Hr1a	6	6	6	6	6	6	6	6	4	4	ID	0	0	2	2	2	18	18	19	19	18	18	18	225	221	222	212	212	222	220	161	165	161	169	193	195
H. casmirica H4	2	2	2	2	2	2	2	2	0	ID	4	4	4	2	2	2	18	18	19	19	16	16	16	224	220	221	211	211	221	220	157	162	159	166	192	191
H. casmirica HM	2	2	2	2	2	2	2	2	ID	0	4	4	4	2	2	2	18	18	19	19	16	16	16	224	220	221	211	211	221	220	157	162	159	166	192	191
H. bacteriophora TT01	0	0	0	0	0	0	0	ID	2	2	6	6	6	4	4	4	20	20	21	21	18	18	18	222	218	219	209	209	221	220	156	161	158	165	191	190
H. bacteriophora HP88	0	0	0	0	0	0	ID	0	2	2	6	6	6	4	4	4	20	20	21	21	18	18	18	222	218	219	209	209	221	220	156	161	158	165	191	190
H. bacteriophora Brecon	0	0	0	0	0	ID	0	0	2	2	6	6	6	4	4	4	20	20	21	21	18	18	18	222	218	219	209	209	221	220	156	161	158	165	191	190
H. bacteriophora DE2	0	0	0	0	ID	0	0	0	2	2	6	6	6	4	4	4	20	20	21	21	18	18	18	222	218	219	209	209	221	220	156	161	158	165	191	190
H. bacteriophora DE6	0	0	0	ID	0	0	0	0	2	2	6	6	6	4	4	4	20	20	21	21	18	18	18	222	218	219	209	209	221	220	156	161	158	165	191	190
H. bacteriophora IT6	0	0	ID	0	0	0	0	0	2	2	6	6	6	4	4	4	20	20	21	21	18	18	18	222	218	219	209	209	221	220	156	161	158	165	191	190
H. bacteriophora EN01	0	ID	0	0	0	0	0	0	2	2	6	6	6	4	4	4	20	20	21	21	18	18	18	222	218	219	209	209	221	220	156	161	158	165	191	190
H. bacteriophora PT1	ID	0	0	0	0	0	0	0	2	2	6	6	6	4	4	4	20	20	21	21	18	18	18	222	218	219	209	209	221	220	156	161	158	165	191	190

Figure S5. Pairwise comparisons of the number of nucleotide differences in the sequences of the internal transcribed spacer (ITS) region of the rRNA gene of different *Heterorhabditis* species. A total of 765 nucleotide positions, flanked by primers TW81 and AB28, were analyzed. Accession numbers of gene sequences used are shown in Table S2.

	H. bacteriophora EN01	*H. bacteriophora* PT1	*H. bacteriophora* IT6	*H. bacteriophora* DE6	*H. bacteriophora* DE2	*H. bacteriophora* TT01	*H. bacteriophora* Brecon	*H. bacteriophora* HP88	*H. casmirica* H4	*H. casmirica* HM	*H. zacatecana* MEX-41	*H. zacatecana* MEX-40	*H. zacatecana* MEX-39	*H. ruandica* Rw18_M-Hr1b	*H. ruandica* Rw18_M-Hr1a	*H. ruandica* Rw14_N-C4a	*H. georgiana* Kesha	*H. georgiana* Hbb	***H. americana* n. sp. S10**	***H. americana* n. sp. S8**	*H. beicherriana* Cherry	*H. beicherriana* M6	*H. beicherriana* CD2516	*H. taysearae* Benin	*H. floridensis* 332	*H. mexicana* MX	*H. baujardi* CD2519	*H. amazonensis* CD2510	*H. noenieputensis* SF669	*H. indica* MEX10	*H. safricana* SF281	*H. marelatus*	*H. atacamensis* MEX-20	*H. downesi* CD2508	*H. megidis* CD2518	*H. zealandica* CD2507
H. zealandica CD2507	94.8	94.8	94.8	94.8	94.8	94.8	94.8	94.8	94.8	94.8	95.0	95.0	95.0	95.0	95.0	95.0	94.6	94.6	94.6	94.6	95.0	95.0	95.0	91.2	91.2	91.4	91.6	91.8	90.5	90.7	97.9	97.9	98.5	98.7	97.6	ID
H. megidis CD2518	93.7	93.7	93.7	93.7	93.7	93.7	93.7	93.7	93.7	93.7	93.9	93.9	93.9	93.9	93.9	93.9	93.5	93.5	93.5	93.5	93.9	93.9	93.9	90.4	90.4	90.5	90.7	90.9	90.0	90.2	97.4	97.4	97.9	98.1	ID	97.6
H. downesi CD2508	95.4	95.4	95.4	95.4	95.4	95.4	95.4	95.4	95.4	95.4	95.5	95.5	95.5	95.5	95.5	95.5	95.2	95.2	95.2	95.2	95.5	95.5	95.5	92.1	92.1	92.3	92.5	92.7	91.4	91.6	99.2	99.4	99.8	ID	98.1	98.7
H. atacamensis MEX-20	95.5	95.5	95.5	95.5	95.5	95.5	95.5	95.5	95.5	95.5	95.7	95.7	95.7	95.7	95.7	95.7	95.4	95.4	95.4	95.4	95.7	95.7	95.7	92.0	92.0	92.1	92.3	92.5	91.6	91.8	99.4	99.4	ID	99.8	97.9	98.5
H. marelatus	95.0	95.0	95.0	95.0	95.0	95.0	95.0	95.0	95.0	95.0	95.2	95.2	95.2	95.2	95.2	95.2	94.8	94.8	94.8	94.8	95.2	95.2	95.2	91.4	91.4	91.6	91.8	92.0	91.1	91.2	98.8	ID	99.4	99.2	97.4	97.9
H. safricana SF281	95.0	95.0	95.0	95.0	95.0	95.0	95.0	95.0	95.0	95.0	95.2	95.2	95.2	95.2	95.2	95.2	94.8	94.8	94.8	94.8	95.2	95.2	95.2	91.6	91.6	91.8	92.0	92.1	92.0	92.1	ID	98.8	99.4	99.2	97.4	97.9
H. indica LN2	91.2	91.2	91.2	91.2	91.2	91.2	91.2	91.2	91.2	91.2	91.4	91.4	91.4	91.4	91.4	91.4	91.1	91.1	91.1	91.1	91.4	91.4	91.4	95.6	95.8	95.8	96.1	96.1	99.6	ID	92.1	91.2	91.8	91.6	90.2	90.7
H. noenieputensis SF669	91.1	91.1	91.1	91.1	91.1	91.1	91.1	91.1	91.1	91.1	91.3	91.3	91.3	91.3	91.3	91.3	90.9	90.9	90.9	90.9	91.3	91.3	91.3	95.6	95.8	95.8	96.1	96.1	ID	99.6	92.0	91.1	91.6	91.4	90.0	90.5
H. amazonensis CD2510	92.3	92.3	92.3	92.3	92.3	92.3	92.3	92.3	92.3	92.3	92.1	92.1	92.1	92.1	92.1	92.1	91.8	91.8	91.8	91.8	92.1	92.1	92.1	98.9	98.9	98.7	99.8	ID	96.1	96.1	92.1	92.0	92.5	92.7	90.9	91.8
H. baujardi CD2519	92.1	92.1	92.1	92.1	92.1	92.1	92.1	92.1	92.1	92.1	92.0	92.0	92.0	92.0	92.0	92.0	91.6	91.6	91.6	91.6	92.0	92.0	92.0	98.9	98.9	98.7	ID	99.8	96.1	96.1	92.0	91.8	92.3	92.5	90.7	91.6
H. mexicana MX	92.0	92.0	92.0	92.0	92.0	92.0	92.0	92.0	92.0	92.0	91.8	91.8	91.8	91.8	91.8	91.8	91.4	91.4	91.4	91.4	91.8	91.8	91.8	99.8	99.0	ID	98.7	98.7	95.8	95.8	91.8	91.6	92.1	92.3	90.5	91.4
H. floridensis 332	92.1	92.1	92.1	92.1	92.1	92.1	92.1	92.1	92.1	92.1	92.0	92.0	92.0	92.0	92.0	92.0	91.6	91.6	91.6	91.6	92.0	92.0	92.0	99.2	ID	99.0	98.9	98.9	95.8	95.8	91.6	91.4	92.0	92.1	90.4	91.2
H. taysearae Benin	91.8	91.8	91.8	91.8	91.8	91.8	91.8	91.8	91.8	91.8	91.6	91.6	91.6	91.6	91.6	91.6	91.2	91.2	91.2	91.2	91.6	91.6	91.6	ID	99.2	99.8	98.9	98.9	95.6	95.6	91.6	91.4	92.0	92.1	90.4	91.2
H. beicherriana CD2516	99.4	99.4	99.4	99.4	99.4	99.4	99.4	99.4	99.4	99.4	99.6	99.6	99.6	99.6	99.6	99.6	99.6	99.6	99.6	99.6	100	100	ID	91.6	92.0	91.8	92.0	92.1	91.3	91.4	95.2	95.2	95.7	95.5	93.9	95.0
H. beicherriana M6	99.4	99.4	99.4	99.4	99.4	99.4	99.4	99.4	99.4	99.4	99.6	99.6	99.6	99.6	99.6	99.6	99.6	99.6	99.6	99.6	100	ID	100	91.6	92.0	91.8	92.0	92.1	91.3	91.4	95.2	95.2	95.7	95.5	93.9	95.0
H. beicherriana Cherry	99.4	99.4	99.4	99.4	99.4	99.4	99.4	99.4	99.4	99.4	99.6	99.6	99.6	99.6	99.6	99.6	99.6	99.6	99.6	99.6	ID	100	100	91.6	92.0	91.8	92.0	92.1	91.3	91.4	95.2	95.2	95.7	95.5	93.9	95.0
***H. americana* n. sp. S8**	99.4	99.4	99.4	99.4	99.4	99.4	99.4	99.4	99.4	99.4	99.6	99.6	99.6	99.6	99.6	99.6	100	100	100	ID	99.6	99.6	99.6	91.2	91.6	91.4	91.6	91.8	90.9	91.1	94.8	94.8	95.4	95.2	93.5	94.6
***H. americana* n. sp. S10**	99.4	99.4	99.4	99.4	99.4	99.4	99.4	99.4	99.4	99.4	99.6	99.6	99.6	99.6	99.6	99.6	100	100	ID	100	99.6	99.6	99.6	91.2	91.6	91.4	91.6	91.8	90.9	91.1	94.8	94.8	95.4	95.2	93.5	94.6
H. georgiana Hbb	99.4	99.4	99.4	99.4	99.4	99.4	99.4	99.4	99.4	99.4	99.6	99.6	99.6	99.6	99.6	99.6	100	ID	100	100	99.6	99.6	99.6	91.2	91.6	91.4	91.6	91.8	90.9	91.1	94.8	94.8	95.4	95.2	93.5	94.6
H. georgiana Kesha	99.4	99.4	99.4	99.4	99.4	99.4	99.4	99.4	99.4	99.4	99.6	99.6	99.6	99.6	99.6	99.6	ID	100	100	100	99.6	99.6	99.6	91.2	91.6	91.4	91.6	91.8	90.9	91.1	94.8	94.8	95.4	95.2	93.5	94.6
H. ruandica Rw14_N-C4a	99.8	99.8	99.8	99.8	99.8	99.8	99.8	99.8	99.8	99.8	100	100	100	100	100	ID	99.6	99.6	99.6	99.6	99.6	99.6	99.6	91.6	92.0	91.8	92.0	92.1	91.3	91.4	95.2	95.2	95.7	95.5	93.9	95.0
H. ruandica Rw18_M-Hr1a	99.8	99.8	99.8	99.8	99.8	99.8	99.8	99.8	99.8	99.8	100	100	100	100	ID	100	99.6	99.6	99.6	99.6	99.6	99.6	99.6	91.6	92.0	91.8	92.0	92.1	91.3	91.4	95.2	95.2	95.7	95.5	93.9	95.0
H. ruandica Rw18_M-Hr1b	99.8	99.8	99.8	99.8	99.8	99.8	99.8	99.8	99.8	99.8	100	100	100	ID	100	100	99.6	99.6	99.6	99.6	99.6	99.6	99.6	91.6	92.0	91.8	92.0	92.1	91.3	91.4	95.2	95.2	95.7	95.5	93.9	95.0
H. zacatecana MEX-39	99.8	99.8	99.8	99.8	99.8	99.8	99.8	99.8	99.8	99.8	100	100	ID	100	100	100	99.6	99.6	99.6	99.6	99.6	99.6	99.6	91.6	92.0	91.8	92.0	92.1	91.3	91.4	95.2	95.2	95.7	95.5	93.9	95.0
H. zacatecana MEX-40	99.8	99.8	99.8	99.8	99.8	99.8	99.8	99.8	99.8	99.8	100	ID	100	100	100	100	99.6	99.6	99.6	99.6	99.6	99.6	99.6	91.6	92.0	91.8	92.0	92.1	91.3	91.4	95.2	95.2	95.7	95.5	93.9	95.0
H. zacatecana MEX-41	99.8	99.8	99.8	99.8	99.8	99.8	99.8	99.8	99.8	99.8	ID	100	100	100	100	100	99.6	99.6	99.6	99.6	99.6	99.6	99.6	91.6	92.0	91.8	92.0	92.1	91.3	91.4	95.2	95.2	95.7	95.5	93.9	95.0
H. casmirica HM	100	100	100	100	100	100	100	100	100	ID	99.8	99.8	99.8	99.8	99.8	99.8	99.4	99.4	99.4	99.4	99.4	99.4	99.4	91.8	92.1	92.0	92.1	92.3	91.1	91.2	95.0	95.0	95.5	95.4	93.7	94.8
H. casmirica H4	100	100	100	100	100	100	100	100	ID	100	99.8	99.8	99.8	99.8	99.8	99.8	99.4	99.4	99.4	99.4	99.4	99.4	99.4	91.8	92.1	92.0	92.1	92.3	91.1	91.2	95.0	95.0	95.5	95.4	93.7	94.8
H. bacteriophora HP88	100	100	100	100	100	100	100	ID	100	100	99.8	99.8	99.8	99.8	99.8	99.8	99.4	99.4	99.4	99.4	99.4	99.4	99.4	91.8	92.1	92.0	92.1	92.3	91.1	91.2	95.0	95.0	95.5	95.4	93.7	94.8
H. bacteriophora Brecon	100	100	100	100	100	100	ID	100	100	100	99.8	99.8	99.8	99.8	99.8	99.8	99.4	99.4	99.4	99.4	99.4	99.4	99.4	91.8	92.1	92.0	92.1	92.3	91.1	91.2	95.0	95.0	95.5	95.4	93.7	94.8
H. bacteriophora TT01	100	100	100	100	100	ID	100	100	100	100	99.8	99.8	99.8	99.8	99.8	99.8	99.4	99.4	99.4	99.4	99.4	99.4	99.4	91.8	92.1	92.0	92.1	92.3	91.1	91.2	95.0	95.0	95.5	95.4	93.7	94.8
H. bacteriophora DE2	100	100	100	100	ID	100	100	100	100	100	99.8	99.8	99.8	99.8	99.8	99.8	99.4	99.4	99.4	99.4	99.4	99.4	99.4	91.8	92.1	92.0	92.1	92.3	91.1	91.2	95.0	95.0	95.5	95.4	93.7	94.8
H. bacteriophora DE6	100	100	100	ID	100	100	100	100	100	100	99.8	99.8	99.8	99.8	99.8	99.8	99.4	99.4	99.4	99.4	99.4	99.4	99.4	91.8	92.1	92.0	92.1	92.3	91.1	91.2	95.0	95.0	95.5	95.4	93.7	94.8
H. bacteriophora IT6	100	100	ID	100	100	100	100	100	100	100	99.8	99.8	99.8	99.8	99.8	99.8	99.4	99.4	99.4	99.4	99.4	99.4	99.4	91.8	92.1	92.0	92.1	92.3	91.1	91.2	95.0	95.0	95.5	95.4	93.7	94.8
H. bacteriophora PT1	100	ID	100	100	100	100	100	100	100	100	99.8	99.8	99.8	99.8	99.8	99.8	99.4	99.4	99.4	99.4	99.4	99.4	99.4	91.8	92.1	92.0	92.1	92.3	91.1	91.2	95.0	95.0	95.5	95.4	93.7	94.8
H. bacteriophora EN01	ID	100	100	100	100	100	100	100	100	100	99.8	99.8	99.8	99.8	99.8	99.8	99.4	99.4	99.4	99.4	99.4	99.4	99.4	91.8	92.1	92.0	92.1	92.3	91.1	91.2	95.0	95.0	95.5	95.4	93.7	94.8

Figure S6. Pairwise comparisons of the nucleotide similarity (%) values for the sequences of the D2-D3 expansion segments of the 28S rRNA gene of different *Heterorhabditis* species. A total of 554 nucleotide positions, flanked by primers D2A and D3B, were analyzed. Accession numbers of gene sequences used for the analyses are shown in Table S2.

	H. bacteriophora EN01	*H. bacteriophora* PT1	*H. bacteriophora* IT6	*H. bacteriophora* DE6	*H. bacteriophora* DE2	*H. bacteriophora* TT01	*H. bacteriophora* Brecon	*H. bacteriophora* HP88	*H. casmirica* H4	*H. casmirica* HM	*H. zacatecana* MEX-41	*H. zacatecana* MEX-40	*H. zacatecana* MEX-39	*H. ruandica* Rw18_M-Hr1b	*H. ruandica* Rw18_M-Hr1a	*H. ruandica* Rw14_N-C4a	*H. georgiana* Kesha	*H. georgiana* Hbb	***H. americana* n. sp. S10**	***H. americana* n. sp. S8**	*H. beicherriana* Cherry	*H. beicherriana* M6	*H. beicherriana* CD2516	*H. taysearae* Benin	*H. floridensis* 332	*H. mexicana* MX	*H. baujardi* CD2519	*H. amazonensis* CD2510	*H. noenieputensis* SF669	*H. indica* LN2	*H. safricana* SF281	*H. marelatus*	*H. atacamensis* MEX-20	*H. downesi* CD2508	*H. megidis* CD2518	*H. zealandica* CD2507
H. zealandica CD2507	28	28	28	28	28	28	28	28	28	28	27	27	27	27	27	27	29	29	29	29	27	27	27	48	48	47	46	45	52	51	11	11	8	7	13	ID
H. megidis CD2518	34	34	34	34	34	34	34	34	34	34	33	33	33	33	33	33	35	35	35	35	33	33	33	53	53	52	51	50	55	54	14	14	11	10	ID	13
H. downesi CD2508	25	25	25	25	25	25	25	25	25	25	24	24	24	24	24	24	26	26	26	26	24	24	24	43	43	42	41	40	47	46	4	4	1	ID	10	7
H. atacamensis MEX-20	24	24	24	24	24	24	24	24	24	24	23	23	23	23	23	23	25	25	25	25	23	23	23	44	44	43	42	41	46	45	3	3	ID	1	11	8
H. marelatus	27	27	27	27	27	27	27	27	27	27	26	26	26	26	26	26	28	28	28	28	26	26	26	47	47	46	45	44	49	48	6	ID	3	4	14	11
H. safricana SF281	27	27	27	27	27	27	27	27	27	27	26	26	26	26	26	26	28	28	28	28	26	26	26	46	46	45	44	43	44	43	ID	6	3	4	14	11
H. indica LN2	48	48	48	48	48	48	48	48	48	48	47	47	47	47	47	47	49	49	49	49	47	47	47	24	23	23	21	21	2	ID	43	48	45	46	54	51
H. noenieputensis SF669	49	49	49	49	49	49	49	49	49	49	48	48	48	48	48	48	50	50	50	50	48	48	48	24	23	23	21	21	ID	2	44	49	46	47	55	52
H. amazonensis CD2510	42	42	42	42	42	42	42	42	42	42	43	43	43	43	43	43	45	45	45	45	43	43	43	6	6	7	1	ID	21	21	43	44	41	40	50	45
H. baujardi CD2519	43	43	43	43	43	43	43	43	43	43	44	44	44	44	44	44	46	46	46	46	44	44	44	6	6	7	ID	1	21	21	44	45	42	41	51	46
H. mexicana MX	44	44	44	44	44	44	44	44	44	44	45	45	45	45	45	45	47	47	47	47	45	45	45	1	5	ID	7	7	23	23	45	46	43	42	52	47
H. floridensis 332	43	43	43	43	43	43	43	43	43	43	44	44	44	44	44	44	46	46	46	46	44	44	44	4	ID	5	6	6	23	23	46	47	44	43	53	48
H. taysearae Benin	45	45	45	45	45	45	45	45	45	45	46	46	46	46	46	46	48	48	48	48	46	46	46	ID	4	1	6	6	24	24	46	47	44	43	53	48
H. beicherriana CD2516	3	3	3	3	3	3	3	3	3	3	2	2	2	2	2	2	2	2	2	2	0	0	ID	46	44	45	44	43	48	47	26	26	23	24	33	27
H. beicherriana M6	3	3	3	3	3	3	3	3	3	3	2	2	2	2	2	2	2	2	2	2	0	ID	0	46	44	45	44	43	48	47	26	26	23	24	33	27
H. beicherriana Cherry	3	3	3	3	3	3	3	3	3	3	2	2	2	2	2	2	2	2	2	2	ID	0	0	46	44	45	44	43	48	47	26	26	23	24	33	27
***H. americana* n. sp. S8**	3	3	3	3	3	3	3	3	3	3	2	2	2	2	2	2	0	0	0	ID	2	2	2	48	46	47	46	45	50	49	28	28	25	26	35	29
***H. americana* n. sp. S10**	3	3	3	3	3	3	3	3	3	3	2	2	2	2	2	2	0	0	ID	0	2	2	2	48	46	47	46	45	50	49	28	28	25	26	35	29
H. georgiana Hbb	3	3	3	3	3	3	3	3	3	3	2	2	2	2	2	2	0	ID	0	0	2	2	2	48	46	47	46	45	50	49	28	28	25	26	35	29
H. georgiana Kesha	3	3	3	3	3	3	3	3	3	3	2	2	2	2	2	2	ID	0	0	0	2	2	2	48	46	47	46	45	50	49	28	28	25	26	35	29
H. ruandica Rw14_N-C4a	1	1	1	1	1	1	1	1	1	1	0	0	0	0	0	ID	2	2	2	2	2	2	2	46	44	45	44	43	48	47	26	26	23	24	33	27
H. ruandica Rw18_M-Hr1a	1	1	1	1	1	1	1	1	1	1	0	0	0	0	ID	0	2	2	2	2	2	2	2	46	44	45	44	43	48	47	26	26	23	24	33	27
H. ruandica Rw18_M-Hr1b	1	1	1	1	1	1	1	1	1	1	0	0	0	ID	0	0	2	2	2	2	2	2	2	46	44	45	44	43	48	47	26	26	23	24	33	27
H. zacatecana MEX-39	1	1	1	1	1	1	1	1	1	1	0	0	ID	0	0	0	2	2	2	2	2	2	2	46	44	45	44	43	48	47	26	26	23	24	33	27
H. zacatecana MEX-40	1	1	1	1	1	1	1	1	1	1	0	ID	0	0	0	0	2	2	2	2	2	2	2	46	44	45	44	43	48	47	26	26	23	24	33	27
H. zacatecana MEX-41	1	1	1	1	1	1	1	1	1	1	ID	0	0	0	0	0	2	2	2	2	2	2	2	46	44	45	44	43	48	47	26	26	23	24	33	27
H. casmirica HM	0	0	0	0	0	0	0	0	0	ID	1	1	1	1	1	1	3	3	3	3	3	3	3	45	43	44	43	42	49	48	27	27	24	25	34	28
H. casmirica H4	0	0	0	0	0	0	0	0	ID	0	1	1	1	1	1	1	3	3	3	3	3	3	3	45	43	44	43	42	49	48	27	27	24	25	34	28
H. bacteriophora HP88	0	0	0	0	0	0	0	ID	0	0	1	1	1	1	1	1	3	3	3	3	3	3	3	45	43	44	43	42	49	48	27	27	24	25	34	28
H. bacteriophora Brecon	0	0	0	0	0	0	ID	0	0	0	1	1	1	1	1	1	3	3	3	3	3	3	3	45	43	44	43	42	49	48	27	27	24	25	34	28
H. bacteriophora TT01	0	0	0	0	0	ID	0	0	0	0	1	1	1	1	1	1	3	3	3	3	3	3	3	45	43	44	43	42	49	48	27	27	24	25	34	28
H. bacteriophora DE2	0	0	0	0	ID	0	0	0	0	0	1	1	1	1	1	1	3	3	3	3	3	3	3	45	43	44	43	42	49	48	27	27	24	25	34	28
H. bacteriophora DE6	0	0	0	ID	0	0	0	0	0	0	1	1	1	1	1	1	3	3	3	3	3	3	3	45	43	44	43	42	49	48	27	27	24	25	34	28
H. bacteriophora IT6	0	0	ID	0	0	0	0	0	0	0	1	1	1	1	1	1	3	3	3	3	3	3	3	45	43	44	43	42	49	48	27	27	24	25	34	28
H. bacteriophora PT1	0	ID	0	0	0	0	0	0	0	0	1	1	1	1	1	1	3	3	3	3	3	3	3	45	43	44	43	42	49	48	27	27	24	25	34	28
H. bacteriophora EN01	ID	0	0	0	0	0	0	0	0	0	1	1	1	1	1	1	3	3	3	3	3	3	3	45	43	44	43	42	49	48	27	27	24	25	34	28

Figure S7. Pairwise comparisons of the number of nucleotide differences in the sequences of the D2-D3 expansion segments of the 28S rRNA gene of different *Heterorhabditis* species. A total of 554 nucleotide positions, flanked by primers D2A and D3B, were analyzed. Accession numbers of gene sequences used for the analyses are shown in Table S2.

	P. asymbiotica DSM 15149^T	*P. australis* subsp. *australis* DSM 17609^T	*P. australis* subsp. *thailandensis* PB68.1^T	*P. cinerea* DSM 19724^T	*P. heterorhabditis* subsp. *heterorhabditis* SF41^T	*P. heterorhabditis* subsp. *aluminescens* Q614^T	*P. tasmaniensis* DSM 22387^T	*P. thracensis* DSM 15199^T	*P. temperata* DSM 14550^T	*P. stackebrandtii* DSM 23271^T	*P. khanii* subsp. *khanii* DSM 3369^T	*P. khanii* subsp. *guanajuatensis* MEX20-17^T	*P. laumondii* subsp. *laumondii* TT01^T	*P. laumondii* subsp. *clarkei* BOJ-47^T	*P. kayaii* DSM 15194^T	*P. bodei* LJ24-63^T	*P. kleinii* DSM 23513^T	***P. kleinii* S8**	***P. kleinii* S10**	*P. luminescence* subsp. *luminescence* ATCC 29999^T	*P. luminescens* subsp. *mexicana* MEX47-22^T	*P. noenieputensis* DSM 25462^T	*P. caribbeanensis* HG29^T	*P. aegyptia* BA1^T	*P. akhurstii* subsp. *akhurstii* DSM 15138^T	*P. akhurstii* subsp. *bharatensis* H3^T	*P. hindustanensis* H1^T	*P. hainanensis* DSM 22397^T	*P. namnaonensis* PB45.5^T
P. asymbiotica DSM 15149^T	ID	49	49	43	43	42	31	32	31	32	31	31	31	31	31	31	31	31	31	31	31	30	30	30	30	30	30	30	30
P. australis subsp. *australis* DSM 17609^T	49	ID	77	44	44	45	31	32	31	31	31	31	31	31	31	32	31	31	31	30	30	31	30	31	31	31	31	31	30
P. australis subsp. *thailandensis* PB68.1^T	49	77	ID	44	44	44	31	32	31	31	31	31	31	31	32	32	31	31	31	30	30	31	30	31	31	31	31	31	31
P. cinerea DSM 19724^T	43	44	44	ID	58	59	32	33	33	33	32	32	32	31	32	32	31	31	31	30	30	31	30	31	31	31	30	31	30
P. heterorhabditis subsp. *heterorhabditis* SF41^T	43	44	44	58	ID	75	32	33	32	32	32	32	32	32	32	32	32	32	32	31	30	32	31	31	31	31	31	31	31
P. heterorhabditis subsp. *aluminescens* Q614^T	42	45	44	59	75	ID	33	33	33	32	32	32	32	32	32	33	32	32	32	31	30	31	31	31	31	31	30	31	31
P. tasmaniensis DSM 22387^T	31	31	31	32	32	33	ID	51	51	48	48	48	32	32	33	33	32	32	32	31	31	31	31	32	31	31	31	31	31
P. thracensis DSM 15199^T	32	32	32	33	33	33	51	ID	67	53	52	52	33	33	35	34	34	34	34	32	32	32	32	32	32	32	32	32	32
P. temperata DSM 14550^T	31	31	31	33	32	33	51	67	ID	53	52	52	32	32	33	33	32	33	33	31	31	31	31	32	31	31	31	31	31
P. stackebrandtii DSM 23271^T	32	31	31	33	32	32	48	53	53	ID	64	57	33	33	33	33	34	34	34	32	32	32	31	32	31	31	31	31	31
P. khanii subsp. *khanii* DSM 3369^T	31	31	31	32	32	32	48	52	52	64	ID	70	32	33	33	33	33	33	33	32	32	31	31	32	31	31	31	31	31
P. khanii subsp. *guanajuatensis* MEX20-17^T	31	31	31	32	32	32	48	52	52	57	70	ID	32	32	33	33	33	33	33	32	32	32	31	32	31	31	31	31	31
P. laumondii subsp. *laumondii* TT01^T	31	31	31	32	32	32	32	33	32	33	32	32	ID	77	58	58	53	53	53	44	44	49	47	47	46	46	45	46	45
P. laumondii subsp. *clarkei* BOJ-47^T	31	31	31	31	32	32	32	33	32	33	33	32	77	ID	59	60	54	55	55	44	45	48	47	48	48	48	47	48	46
P. kayaii DSM 15194^T	31	31	32	32	32	32	33	35	33	33	33	33	58	59	ID	69	64	63	63	45	45	48	47	47	47	47	46	47	46
P. bodei LJ24-63^T	31	32	32	32	32	33	33	34	33	33	33	33	58	60	69	ID	69	69	69	45	45	48	47	48	48	48	48	48	47
P. kleinii DSM 23513^T	31	31	31	31	32	32	32	34	32	34	33	33	53	54	64	69	ID	94	94	48	47	47	46	46	46	46	45	45	45
***P. kleinii* S8**	31	31	31	31	32	32	32	34	33	34	33	33	53	55	63	69	94	ID	100	48	47	47	46	46	46	46	45	45	45
***P. kleinii* S10**	31	31	31	31	32	32	32	34	33	34	33	33	53	55	63	69	94	100	ID	48	47	47	46	46	46	46	45	45	45
P. luminescens subsp. *luminescens* ATCC 29999^T	31	30	30	30	31	31	31	32	31	32	32	32	44	44	45	45	48	48	48	ID	71	46	46	46	45	45	45	45	44
P. luminescens subsp. *mexicana* MEX47-22^T	31	30	30	30	30	30	31	32	31	32	32	32	44	45	45	45	47	47	47	71	ID	46	46	46	45	45	44	44	44
P. noenieputensis DSM 25462^T	30	31	31	31	32	31	31	32	31	32	31	32	49	48	48	48	47	47	47	46	46	ID	57	55	51	51	50	50	48
P. caribbeanensis HG29^T	30	30	30	30	31	31	31	32	31	31	31	31	47	47	47	47	46	46	46	46	46	57	ID	57	52	52	50	51	49
P. aegyptia BA1^T	30	31	31	31	31	31	32	32	32	32	32	32	47	48	47	48	46	46	46	46	46	55	57	ID	69	66	63	61	56
P. akhurstii subsp. *akhurstii* DSM 15138^T	30	31	31	31	31	31	31	32	31	31	31	31	46	48	47	48	46	46	46	45	45	51	52	69	ID	77	71	69	60
P. akhurstii subsp. *bharatensis* H3^T	30	31	31	31	31	31	31	32	31	31	31	31	46	48	47	48	46	46	46	45	45	51	52	66	77	ID	68	69	59
P. hindustanensis H1^T	30	31	31	30	31	30	31	32	31	31	31	31	45	47	46	48	45	45	45	45	44	50	50	63	71	68	ID	67	64
P. hainanensis DSM 22397^T	30	31	31	31	31	31	31	32	31	31	31	31	46	48	47	48	45	45	45	45	44	50	51	61	69	69	67	ID	61
P. namnaonensis PB45.5^T	30	30	31	30	31	31	31	32	31	31	31	31	45	46	46	47	45	45	45	44	44	48	49	56	60	59	64	61	ID

Figure S8 Pairwise comparisons of digital DNA-DNA Hybridization (dDDH) scores (%) of the type strain of different *Photorhabdus* species/subspecies. Accession numbers of gene sequences used are shown in Table S3.

SUPPLEMENTARY TABLES

Table S1. Sequences of the primers used to amplify the genes/genetic regions of different *Heterorhabditis* species used for the phylogenetic analyses in this study.

Genetic region	Primers	Orientation	Sequence 5' to 3'	Description
ITS	TW81	F	GTTTCCGTAGGTGAACCTGC	Joyce et al. (1994)
	AB28	R	ATATGCTTAAGTTCAGCGGGT	
D2-D3	D2A	F	ACAAGTACCGTGAGGGAAAGTTG	Subbotin et al. (2006)
	D3B	R	TCGGAAGGAACCAGCTACTA	
COI	HCF	F	TTACATGATACTTATTATG	Kuwata et al. (2007)
	HCR	R	CTGATAACTGTGACCAAATACATA	

Table S2. National Center for Biotechnology Information (NCBI) accession numbers of the nucleotide sequences used for the phylogenetic analyses in this study. In bold font are the sequences (22 in total) newly generated in this study.

Organism		Genetic Region		
Species	Strain designation (s)	ITS	D2-D3	COI
H. amazonensis	CD2510, MC01	MT372499	MT372502	MT372499
***H. americana* n. sp.**	**S8**	**PQ483104**	OK646639	**PQ483125**
	**S10**	**PQ483105**	MW817559	**PQ483126**
H. atacamensis	MEX-20	MK421485	MW817561	MW817979
H. bacteriophora	Brecon	**PQ483106**	**PQ492375**	**PQ483127**
	DE2	MZ326036	MW817552	MW817970
	DE6	MZ326037	MW817553	MW817971
	EN01	MZ326038	MW817554	MW817972
	IT6	MZ326039	MW817555	MW817973
	PT1	MZ326040	MW817556	MW817974
	TT01	MZ326041	MW817557	MW817975
	CD2504, HP88	MT372491	MT372509	MT373729
H. baujardi	CD2519, F10	MT372500	MT372503	MT373736
H. beicherriana	M6	**PQ483107**	**PQ492376**	**PQ483128**
	CD2516, QZL-2011	MT372490	MT372511	MT373730
	Cherry	**PQ483108**	**PQ492377**	**PQ483129**
H. casmirica	H4	OQ517949	OQ517940	OQ517980
	HM	OQ517947	OQ517936	OQ517975
H. downesi	CD2508, 23.9	MT372494	MT372507	MT373732
H. floridensis	332	**PQ483109**	**PQ492378**	**PQ483130**
H. georgiana	Hbb	MZ326042	MW817558	MW817976
	Kesha	**PQ483110**	**PQ492379**	**PQ483131**
H. indica	CD2525	MT372498	-	-
	MEX-10	-	MK421439	-
	LN2	-	-	AB355853
H. marelatus	OH10	AY321479	-	-
	No information	-	EU100412	-
	No information	-	-	EF043419
H. megidis	CD2518, DV	MT372495	MT372506	MT373733
H. mexicana	Mexican	AY321478	-	-
	MX	-	EU100414	-
	No information	-	-	EF043422
H. noenieputensis	SF669	JN620538	MT372505	MT373728
H. ruandica	Rw18_M-Hr1a	MZ326033	MW817549	MW817967
	Rw18_M-Hr1b	MZ326034	MW817550	MW817968
	Rw14_N-C4a	MZ326035	MW817551	MW817969
H. safricana	SF281	EF488006	EU100416	OR835850
H. taysearae	Benin	**PQ483111**	**PQ492380**	**PQ483132**
H. zacatecana	MEX-39	MZ326030	MW817546	MW817964
	MEX-40	MZ326031	MW817547	MW817965
	MEX-41	MZ326032	MW817548	MW817966
H. zealandica	CD2507	MT372493	MT372508	MT373734

Table S3. National Center for Biotechnology Information (NCBI) accession numbers of the genomic sequences of different *Photorhabdus* species used in this study.

	NCBI Accession numbers
P. aegyptia BA1^T	JFGV01
P. akhurstii subsp. *akhurstii* DSM 15138^T	RCWE01
P. akhurstii subsp. *bharatensis* H3^T	PUWU01
P. asymbiotica DSM 15149^T	RBLJ01
P. australis subsp. australis DSM 17609^T	JONO01
P. australis subsp. *thailandensis* PB68.1^T	LOMY01
P. bodei LJ24-63^T	NSCM01
P. caribbeanensis DSM 22391^T	RCWB01
P. cinerea DSM 19724^T	PUJW01
P. hainanensis DSM 22397^T	RCWD01
P. heterorhabditis subsp. *heterorhabditis* SF41^T	RCWA01
P. heterorhabditis subsp. *aluminescens* Q614^T	JABBCS01
P. hindustanensis H1^T	PUWT01
P. kayaii DSM 15194T	JAJAFZ01
P. khanii subsp. *khanii* DSM 3369^T	AYSJ01
P. khanii subsp. *guanajuatensis* MEX20-17^T	PUJY01
P. kleinii DSM 23513^T	JAJAFY01
***P. kleinii* S8-52**	**NSCL01**
***P. kleinii* S10-54**	**NSCJ01**
P. laumondii subsp. *clarkei* BOJ-47^T	NSCI01
P. laumondii subsp. *laumondii* TT01^T	WSFH01
P. luminescens subsp. *luminescens* ATCC 29999^T	FMWJ01
P. luminescens subsp. *mexicana* MEX47-22^T	PUJX01
P. namnaonensis PB45.5^T	LOIC01
P. noenieputensis DSM 25462^T	RCWC01
P. stackebrandtii DSM 23271^T	PUJV01
P. tasmaniensis DSM 22387^T	PUJU01
P. temperata DSM 15550^T	JAJAFX01
P. thracensis DSM 15199^T	CP011104
